# Metabolic Imprint of Poliovirus on Glioblastoma Cells and Its Role in Virus Replication and Cytopathic Activity

**DOI:** 10.3390/ijms26157346

**Published:** 2025-07-30

**Authors:** Martin A. Zenov, Dmitry V. Yanvarev, Olga N. Ivanova, Ekaterina A. Denisova, Mikhail V. Golikov, Artemy P. Fedulov, Roman I. Frykin, Viktoria A. Sarkisova, Dmitry A. Goldstein, Peter M. Chumakov, Anastasia V. Lipatova, Alexander V. Ivanov

**Affiliations:** Engelhardt Institute of Molecular Biology, Russian Academy of Sciences, 119991 Moscow, Russia; martin.zenov@yandex.ru (M.A.Z.);

**Keywords:** oncolytic viruses, glioblastoma, poliovirus, polyamines, metabolism, seahorse, respiration, Plasmax

## Abstract

Poliovirus represents an oncolytic agent for human glioblastoma—one of the most aggressive types of cancer. Since interference of viruses with metabolic and redox pathways is often linked to their pathogenesis, drugs targeting metabolic enzymes are regarded as potential enhancers of oncolysis. Our goal was to reveal an imprint of poliovirus on the metabolism of glioblastoma cell lines and to assess the dependence of the virus on these pathways. Using GC-MS, HPLC, and Seahorse techniques, we show that poliovirus interferes with amino acid, purine and polyamine metabolism, mitochondrial respiration, and glycolysis. However, many of these changes are cell line- and culture medium-dependent. 2-Deoxyglucose, the pharmacologic inhibitor of glycolysis, was shown to enhance the cytopathic effect of poliovirus, pointing to its possible repurposing as an enhancer of oncolysis. Inhibitors of polyamine biosynthesis, pyruvate import into mitochondria, and fatty acid oxidation exhibited antiviral activity, albeit in a cell-dependent manner. We also demonstrate that poliovirus does not interfere with the production of superoxide anions or with levels of H_2_O_2_, showing an absence of oxidative stress during infection. Finally, we showed that a high rate of poliovirus replication is associated with fragmentation of the mitochondrial network, pointing to the significance of these organelles for the virus.

## 1. Introduction

Glioblastoma multiforme (GBM) is one of the most aggressive types of malignant brain tumors, which affects many people today. GBM accounts for 54% of all gliomas and 16% of all brain tumors [1]. In developed countries rates of its occurrence range from 2.05 to 3.69 per 100 persons [2]. This tumor is characterized by spontaneous occurrence and extremely low patient survival: 10 months without treatment and 15–16 months with therapy, indicating an extremely unfavorable prognosis [3,4]. Standard treatment approaches for patients with glioblastoma include surgical removal of tumor tissue, radiation therapy, and immunotherapy [5,6,7].

One of the promising approaches for the treatment of cancer and glioblastomas in particular is the usage of oncolytic viruses [8,9]. The mechanism of action of such viruses combines direct lysis of tumor cells and the recruitment of the immune system. Currently, there are four viruses approved for clinical practice [10], and many others are undergoing clinical trials (e.g., [11]). Enteroviruses, such as coxsackie viruses, reoviruses, and vesicular stomatitis virus, present a group of oncolytic viruses that display promising oncolytic activity [12]. Poliovirus, an RNA virus of the *Picornaviridae* family with a diameter of about 30 nm, is currently being actively studied as an oncolytic. It enters cells through interaction with the CD155 receptor, which is overexpressed on the surface of tumor cells, making them more sensitive to this oncolytic agent [13]. It is noteworthy that not only can wild-type poliovirus be used as an oncolytic agent, but also its genetically engineered variants, such as PVSRIPO [14].

Many viral infections are known to perturb the metabolic and redox status of host cells by enhancing aerobic glycolysis, glutaminolysis, and fatty acid synthesis [15,16,17]. Such changes ensure efficient biosynthesis of components of lipid membranes required for enveloped viruses, provide the viral particle with the necessary components of surface glycoproteins, and supply ATP by rapid production during glycolysis [18,19]. Many viruses also interfere with polyamine, cholesterol, and nucleotide metabolism to ensure efficient replication [20,21,22,23,24]. So, metabolic enzymes can act as targets for the development of antiviral agents [17,20,25,26,27]. Indeed, there are examples of clinical trials of their inhibitors used for the treatment of viral infections (e.g., [28]). Many chronic and acute viral infections also trigger oxidative stress, which contributes to their pathogenesis [29,30,31]. In case of chronic hepatitis viruses, antioxidants demonstrate an ability to suppress inflammation and the development of fibrosis [29].

However, there are almost no data on the possible impact of poliovirus on cell metabolism. The dependence of their replication on specific metabolic pathways of host cells also remains obscure. Few reports suggest that poliovirus replication is suppressed when the cells are maintained on poor nutrient media, while the addition of glucose restores virus titer [32]. Poliovirus was also shown to increase phosphatidylcholine production in infected cells, while knockdown of cellular acyl-CoA synthase leads to the inhibition of its replication [33,34].

So, the first goal of this study was to reveal the imprint of poliovirus on metabolic and redox pathways of host cells and to identify targets for antiviral agents among metabolic enzymes. And since we previously demonstrated that the oncolytic activity of coxsackievirus towards glioma cells can be enhanced by an inhibitor of glycolysis [35], the second goal was to assess if this approach can be applied to poliovirus.

## 2. Results

### 2.1. Kinetics of Poliovirus Replication in Glioblastoma Cells Maintained in Various Media

**Figure 1:**
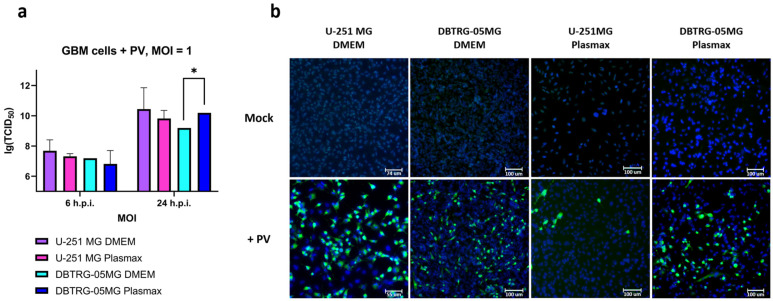
Levels of poliovirus infection in GBM cell lines at 24 h post-infection (h.p.i.). U-251 MG or DBTRG-05MG cell lines maintained in DMEM or Plasmax media were infected with poliovirus at MOI 1 for 6 (**a**) and 24 h (**a**,**b**), and virus infection was monitored by assessment of TCID50 (**a**) or by immunostaining (**b**) using primary antibodies to poliovirus and FITC-conjugated secondary antibodies. TCID50 values (**a**) are presented as means ± SD, * *p* ≤ 0.05 by two-way ANOVA with Tukey post hoc test.

In order to study the impact of poliovirus on the metabolism of GMB cells, it was important to establish when the virus infects a majority of cells but does not yet affect their viability. So, our first task was to evaluate the time course of poliovirus infection in GBM cells cultured either in a conventional nutrient medium (DMEM) or in a plasma-like Plasmax medium. The latter was used as it does not affect cell metabolism itself [36]. We chose two GBM cell lines, namely U-251 MG and DBTRG-05MG. They were maintained either in DMEM supplemented with 10% FBS or Plasmax with 2.5% FBS for at least 6 days, as described previously [37]. It is noteworthy that the medium choice affected cell morphology (Figure S1). Then the cells were infected with type 2 poliovirus at different multiplicities of infection (MOI), and their replication was monitored by the assessment of 50% tissue culture infectious dose (TCID_50_) and by immunofluorescence. We observed an increase in the infectious titer starting from 6 h post-infection (Figure 1a and Figure S2), which correlates with a similar duration of the enterovirus life cycle [38]. In classical DMEM poliovirus replication was more efficient in U-251 MG cells than in DBTRG-05MG cells (Figure S2a,b and Figure 1a). In contrast, no such difference was visible when the cells were maintained in a Plasmax medium (Figure S2c,d and Figure 1a). However, in the U-251 MG cell line, a significantly smaller number of cells were stained positively for the infection (Figure 1b). Nonetheless, in all cases the replication reached a plateau by 24 h.p.i. At this time point PV-induced cell death was minor at MOI 1 in the case of U-251 MG cells (Figure S3) or at MOI 3 in the case of DBTRG-05MG (Figure S4) cells. So we selected 24 h.p.i and the respective MOI for subsequent analysis.

To confirm the successful infection, we performed immunostaining of cells using antibodies to poliovirus (Figure 1b and Figure S5). Indeed, 24 h post-infection almost all cells maintained in DMEM were infected by the virus. Pronounced toxicity in these experiments was noticeable starting from MOI 3 in U-251 MG cells or from MOI 10 in DBTRG-05MG cells (Figure S2). Cultivation of U-251 MG cells in the Plasmax medium resulted in a decrease in infection rates, whereas for DBTRG-05MG cells we observed the opposite effect. So, we confirmed the selection of 24 h.p.i. as the time point for further experiments.

To reveal if different rates of poliovirus infection in these two cell lines in various media were due to different levels of expression of a virus-specific receptor, we quantified the expression of CD155, which is the putative receptor for poliovirus [10]. Substitution of DMEM with Plasmax did not affect CD155 expression in both cell lines (Figure S6a). It indicated that the effect of Plasmax on virus replication was due to other mechanisms.

### 2.2. Changes in Metabolism of Biogenic Polyamines During Poliovirus Infection Are Dispensable for Virus Replication

**Figure 2:**
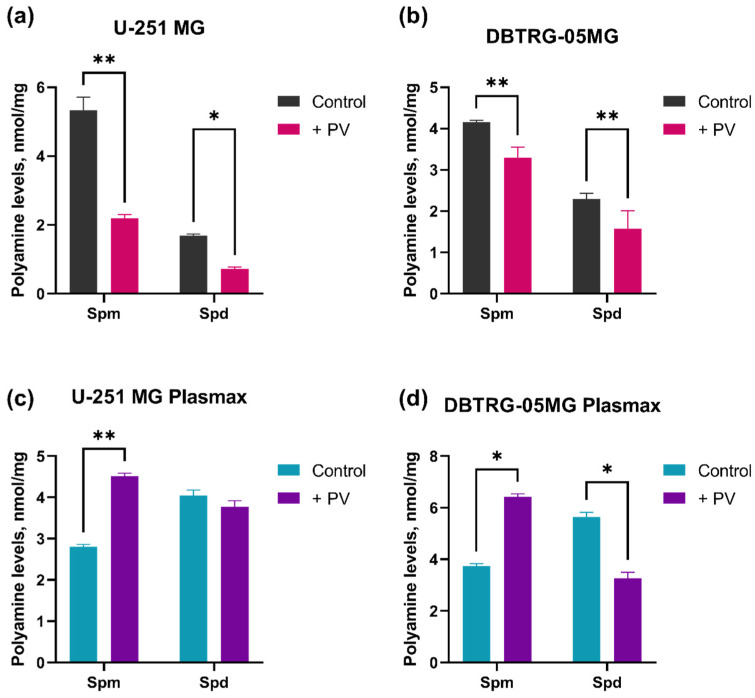
Polyamine levels in GBM cells with and without poliovirus infection, expressed as nmol/mg, measured by high-performance liquid chromatography. U-251 MG (**a**,**c**) and DBTRG-05MG (**b**,**d**) were cultured in DMEM (**a**,**b**) or Plasmax (**c**,**d**) media prior to analysis. Data are presented as mean ± SD, * *p* ≤ 0.05, ** *p* ≤ 0.01 based on two-way ANOVA with Bonferroni test adjustment.

Biogenic polyamines are the ubiquitous compounds indispensable for the replication of various DNA and RNA viruses [39,40]. So, one of our goals was to characterize the impact of poliovirus on their metabolism. Polyamine levels were quantified by HPLC with pre-column derivatization. Poliovirus infection caused a pronounced decrease in spermine and spermidine levels in U-251 MG (Figure 2a) and a moderate decrease in DBTRG-05MG cells (Figure 2b) cultured in DMEM. Both cell lines, when cultured in Plasmax, demonstrated accumulation of spermine upon infection, with a decrease in spermidine in the case of DBTRG-05MG cells (Figure 2c,d). To summarize, the impact of poliovirus on polyamine metabolism depended on the choice of culture medium.

To unveil the importance of polyamines for poliovirus replication and the cytopathogenic effect in GBM cells, we used pharmacological and experimental compounds that inhibit or induce polyamine-metabolizing enzymes. They included (i) difluoromethylornithine (DFMO, Eflornothine^®^—an irreversible inhibitor of cellular ornithine decarboxylase (ODC), (ii) SAM486a (Sardomozide—an inhibitor of S-adenosylmethionine decarboxylase, AMD1), (iii) N^1^,N^4^-bis(2,3-butadienyl)-1,4-butanediamine (MDL 72.527, an inhibitor of spermine oxidase and acetylpolyamine oxidase that mediate polyamine catabolism), (iv) N^1^,N^11^-diethylnorspermine (DENSpm) that induces spermidine/spermine-N^1^-acetyltransferase (SSAT, polyamine catabolism), as well as inhibitors of spermidine and spermine synthases or polyamine-dependent post-translational modification (hypusination) of the eIF5a translation factor (listed in Table 1).

**Figure 3:**
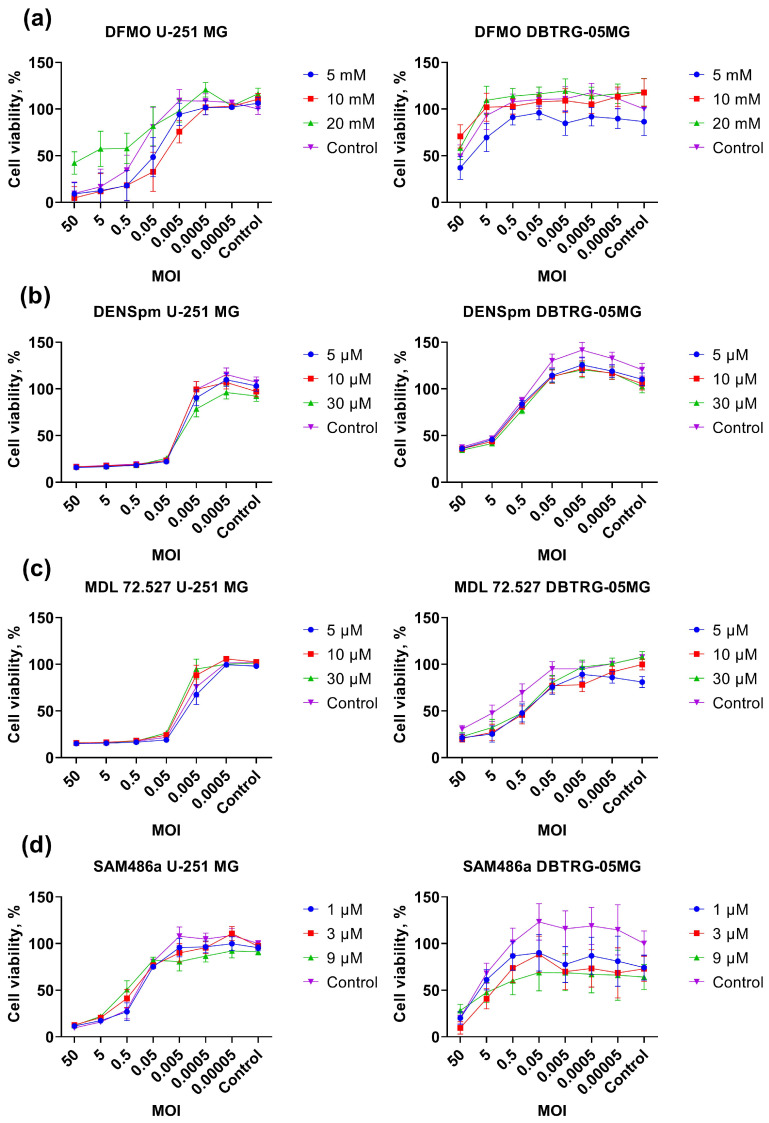
Cytopathogenic effect of poliovirus-infected GBM cells treated with inhibitors or inducers of polyamine-metabolizing enzymes. U-251 MG or DBTRG-05MG cells maintained in DMEM were pretreated with DFMO (**a**), DENSpm (**b**), MDL72.527 (**c**), or SAM486a (**d**), and infected with PV at different MOI in the presence of these compounds at the same concentrations. Cell viability was measured using resazurin assay. The values were normalized to values of untreated mock-infected cells. The data are presented as mean ± SD.

We assessed whether these compounds protected cells from the virus’s cytopathogenic effect (antiviral activity) or enhanced cell death (increased oncolytic activity). As the cell cycle of poliovirus is very short, we added the compounds 24 h before the infection and kept their concentration during the whole experiment. Most of the compounds did not affect cell viability (Figure 3 and Figure S7), showing that poliovirus replication is not dependent on polyamine metabolism. The exceptions were DFMO, which blocks the rate-limiting step of polyamine metabolism (Figure 3a), and deferiprone (DFP, Figure S7a): these compounds partially prevented the death of U-251 MG cells. However, the effect of DFP was likely non-specific, as another compound that targets the same hypusination pathway (GC7) was inactive (Figure S7b). Moreover, the suppression of poliovirus by DFP was probably due to its subtoxic effect, noticeable in uninfected cells. The antiviral activity of DFMO was not confirmed in the DBTRG-05MG cell line (Figure 3a). RT-qPCR analysis revealed that DBTRG-05MG cells are characterized by a lower level of transcription of the DFMO target—ornithine decarboxylase (Figure S6b). Pending the level of ODC mRNA correlates with levels of ODC activity, this would have conferred a higher level of sensitivity of cells to DFMO. So, the absence of anti-poliovirus activity of DFMO in DBTRG-05MG cells could rather be explained by lower rates of infection or by a greater input of polyamine influx into these cells from the culture medium (not quantified in this study). So, poliovirus replication in GBM cells does not rely on most polyamine-metabolizing enzymes, with the exception of DFMO in some cell lines.

### 2.3. Poliovirus Interferes with Central Carbon Metabolism in a Cell Line-Specific Manner

**Figure 4:**
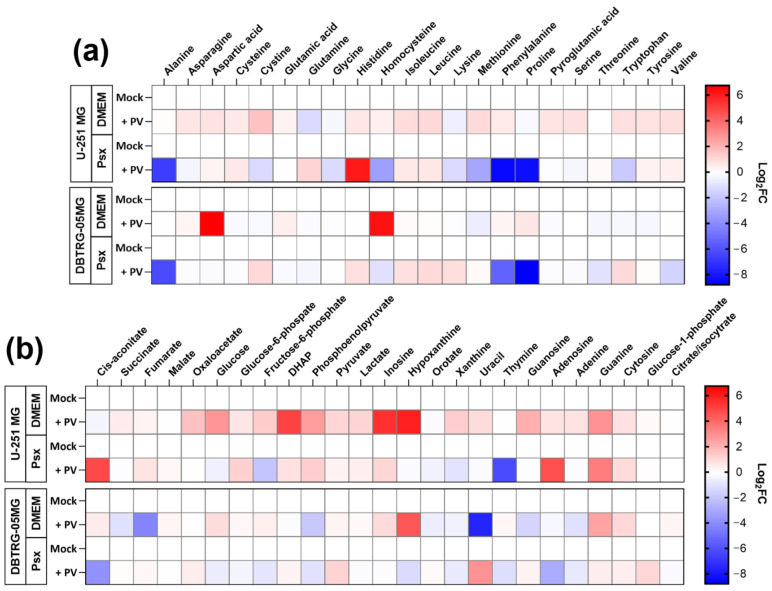
Imprint of poliovirus on levels of polar metabolites of GBM cell lines. U-251 MG or DBTRG-05MG cells maintained in DMEM or Plasmax media were infected with PV at MOI 1, and metabolites levels were measured 24 h.p.i. by gas chromatography-mass spectrometry. The graphs represent amino acids (**a**) and other metabolites (**b**). Levels were normalized to levels in mock-infected cells maintained in respective medium and expressed as log2 fold change (Log_2_FC).

The next step was to evaluate the imprint of poliovirus replication on central carbon metabolism. This was carried out by GC-MS, which allows quantification of all proteinogenic amino acids except arginine, glycolysis, and TCA cycle intermediates, as well as some nucleic bases and nucleosides. These experiments were performed for the same two glioma cell lines maintained in DMEM or Plasmax media. The results are presented in Figure 4. In U-251 MG cells in DMEM, poliovirus caused an increase in most metabolites. The highest upregulation was observed for dihydroxyacetone phosphate (DHAP), a precursor of triglycerides, as well as for purines. These changes suggested upregulation of the respective pathways. However, some of these changes disappeared upon substitution of DMEM with Plasmax, with the exception of increased levels of branched amino acids (Val, Leu, Ile), Tyr, Cys, and Asp, triglycerol precursors (DHAP, glycerol-3-phosphate), most glycolysis intermediates, guanine, adenosine, and inosine. PV infection in U-251 MG cells in Plasmax also resulted in a strong decrease in Ala, Phe, and Pro. In DBTRG-05MG cells the imprint of the virus was different, as most metabolite levels were decreased compared to mock-infected cells. The search for similar changes between two cell lines maintained in a physiological Plasmax medium pointed to the accumulation of His, Leu, Ile, pyruvate, DHAP, and cytosine.

Next, we attempted to reveal genes of glycolysis and triglyceride synthesis that could be responsible for the changes observed in U-251 MG cells. We did not find any impact of poliovirus on the expression of any of the genes, with the exception of AGPS in U-251 MG cells, which is downstream of DHAP (Figure S8). Its induction could not account for the accumulation of this metabolite. We would also like to note a tendency for statistical significance in the case of FASN and TPI mRNA levels (fatty acid synthase and triose phosphate isomerase, responsible for the conversion of DHAP).

### 2.4. Poliovirus Infection Induces Accumulation of Lipid Droplets in GBM Cells

**Figure 5:**
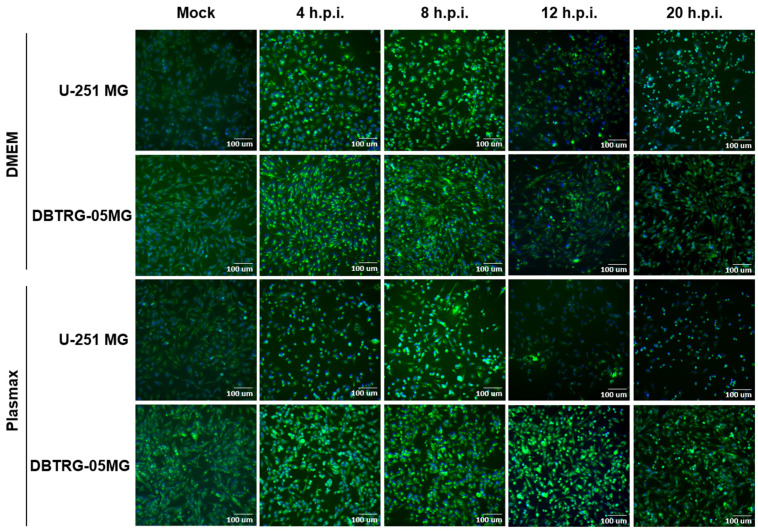
Levels of neutral lipids in GBM cells during poliovirus infection. U-251 MG or DBTRG-05MG cells maintained in DMEM or Plasmax were infected with PV at MOI 1 and stained with BODIPY 493/503 (neutral lipids, green fluorescence) and DAPI (nuclei) at various time points. Fluorescence was visualized on ZOE Fluorescent Cell Imager.

As DHAP and glycerol-3-phosphate are precursors of triglycerides, we assessed levels of neutral lipids using BODIPY 493/503 dye. Four hours post-infection we observed an increase in BODIPY 493/503 fluorescence (Figure 5), which may indicate the accumulation of lipids and their increased biosynthesis during the first cycle of viral replication. Accumulation of neutral lipids reached a plateau by 4–8 h.p.i., whereas upon longer infection the fluorescence levels substantially decreased, except for DBTRG-05MG cells maintained in Plasmax. These results fall in line with previously reported accumulation of neutral lipids followed by their subsequent hydrolysis and conversion into phospholipids in PV-infected HeLa cells [41].

### 2.5. Metabolic Changes in Glioblastoma Cells by Poliovirus Are Not Mediated by Interferon Production

**Figure 6:**
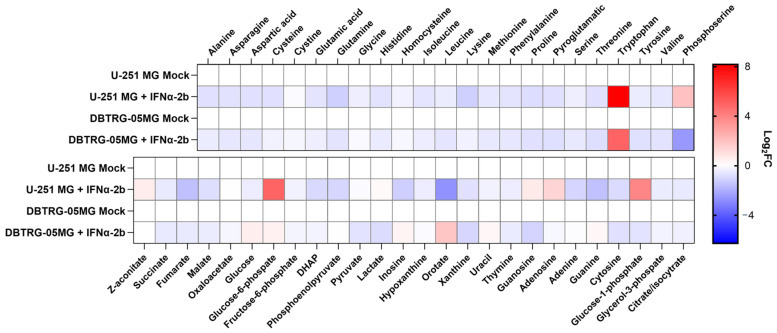
Metabolic changes in GBM cells treated with recombinant interferon α-2b. U-251 MG or DBTRG-05MG cells maintained in DMEM medium were treated with IFN α-2b, and metabolites levels were measured 24 h.p.i. by gas chromatography-mass spectrometry. Levels were normalized to levels in untreated cells and expressed as log_2_ fold change (Log_2_FC).

As RNA viruses trigger interferon production and the concomitant response, the imprint on cell metabolism could be due to this innate immune response, as shown recently for the hepatitis delta virus [42]. So, we evaluated whether metabolic changes were mediated by these antiviral cytokines. First, it was shown that poliovirus infection in U-251 MG cells maintained in DMEM leads to a rather moderate induction of type I and type III interferons (Figure S9). Next, we treated glioma cells with the recombinant IFN α-2b (IFN α-2b activity was verified using real-time PCR, Figure S10) and subjected these cells to metabolic analysis by GC-MS. Interferon induced downregulation of most metabolites in both cell lines (Figure 6). Clearly, there was no correlation between the effect of the cytokine and the virus.

### 2.6. Poliovirus Suppresses Glycolysis and Mitochondrial Respiration

**Figure 7:**
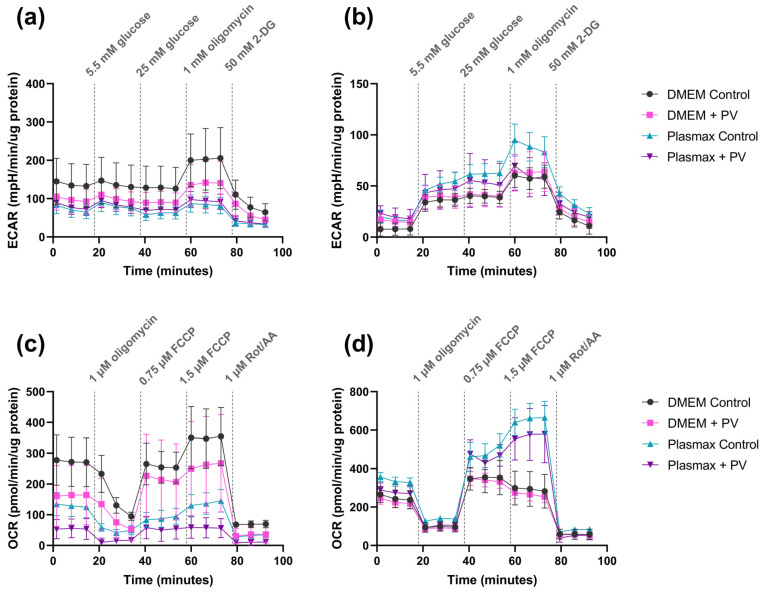
Effect of poliovirus on glycolytic and respiratory activities of GBM cell lines. U-251 MG (**a**,**c**) or DBTRG-05MG (**b**,**d**) cells maintained in DMEM or Plasmax media were infected with PV at MOI 1, and extracellular acidification (ECAR) reflecting glycolysis (**a**,**b**) and oxygen consumption rate (OCR) reflecting respiratory activity (**c**,**d**) were measured by Seahorse technology using GlycoStress and MitoStress assays. Results are presented as mean ± SD.

Our next step was to measure glycolytic and respiratory activities of infected cells to unveil the effect of poliovirus on these two ATP-producing pathways. It was carried out by Seahorse technology, which is the gold standard in this field. Both uninfected cell lines demonstrated similar levels of respiratory activity, though glycolytic activity was much lower in DBTRG-05MG cells than in U-251 MG cells (Figure 7). Poliovirus suppressed glycolysis in U-251 MG cells in DMEM (Figure 7a) and in DBTRG-05MG cells in Plasmax (Figure 7b). The virus also inhibited basal and maximal respiration in U-251 MG cells in both media (Figure 7c), whereas in the second cell line, no changes were observed (Figure 7d). So, the effects of poliovirus infection are cell line-specific.

### 2.7. Efficient Poliovirus Replication in Glioblastoma Cells Requires Fragmented Mitochondria

**Figure 8:**
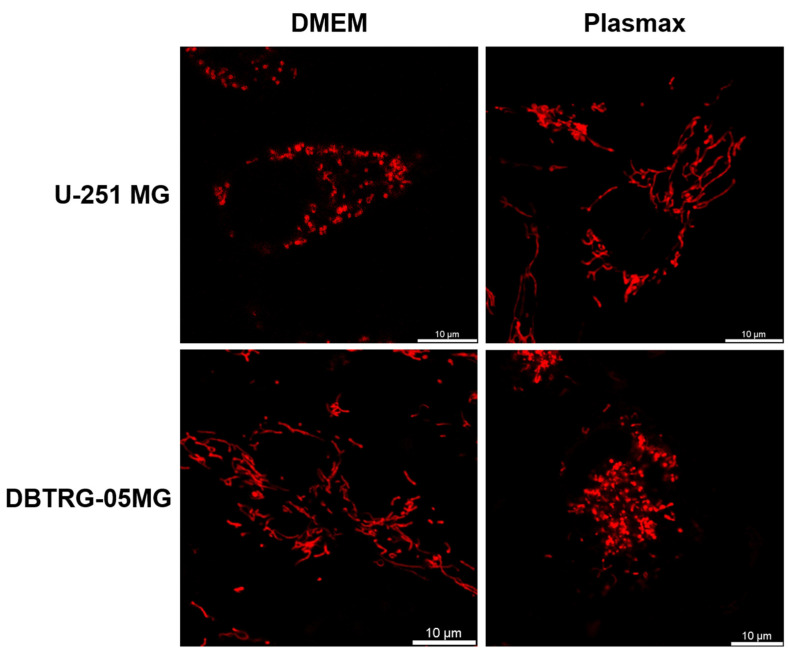
Morphology of mitochondria in U-251 MG and DBTRG-05MG cells maintained in DMEM or Plasmax media. The cells were seeded on confocal dishes; mitochondria were stained with MitoTracker Red and visualized by confocal microscopy. The bar denotes 10 µm.

As respiratory activity depends on the morphology of mitochondria and their fusion into networks, our next step was to evaluate if mitochondria in U-251 MG and DBTRG-05MG cells in DMEM or Plasmax are fragmented or tubular. The uninfected cells were stained with MitoTracker dye, and mitochondrial morphology was assessed by confocal microscopy. In these experiments we did not stain nuclei to prevent the possible toxic effect of DNA-intercalating dyes. The representative images are provided in Figure 8. It shows that in U-251 MG cells maintained in DMEM, as well as in DBTRG-05MG cells in Plasmax (which ensured the highest rates of poliovirus infection), the mitochondria were generally fragmented. In contrast, the change in media that suppressed virus replication triggered the association of mitochondria into vast networks. This indicates that poliovirus replication requires fragmented mitochondria in cells.

### 2.8. Import of Pyruvate into Mitochondria Is Essential for Poliovirus Replication

**Figure 9:**
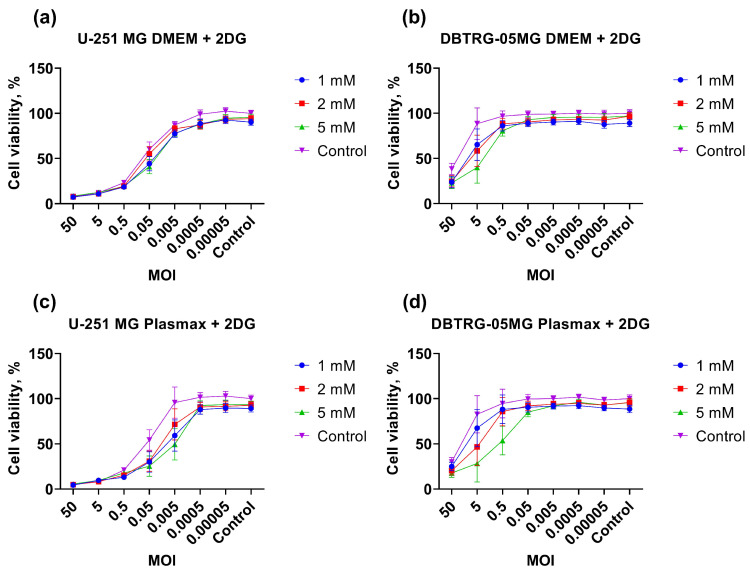
2-deoxyglucose enhances cytopathogenic effect of poliovirus in GBM cells. U-251 MG (**a**,**c**) or DBTRG-05MG (**b**,**d**) cells maintained in DMEM (**a**,**b**) or Plasmax (**c**,**d**) were pretreated with 2-deoxyglucose (2DG) and infected with PV at different MOI in presence of this drug. Cell viability was measured using resazurin assay. Values were normalized to values of untreated mock-infected cells. Data are presented as mean ± SD.

Our next step was to unveil the impact of poliovirus replication on central carbon metabolism using the inhibitors of glycolysis (2-deoxyglucose), mitochondrial pyruvate carrier (UK5099), pyruvate/lactate monocarboxylate transporters (MCTs), fatty acid synthesis (AcSS), β-oxidation (Etomoxir), or mitochondrial respiration (metformin and phenformin). The experiment was carried out as described above for the inhibitors of polyamine metabolism. The results, shown in Figure 9, Figures S11 and S12, demonstrate that poliovirus replication is not dependent on pyruvate or lactate export from cell or fatty acid biosynthesis. At the same time the virus requires the import of pyruvate from the cytoplasm to mitochondria, at least in U-251 MG cells (Figure S12a). Inhibition of fatty acid oxidation-derived acyl-CoA import into mitochondria using Etomoxir suppressed poliovirus replication, albeit in DBTRG-05MG cells, no dependency of the effect on drug concentration was observed (Figure S11a,d). Most importantly, inhibition of glycolysis by the 2DG enhanced the cytopathic effect of the virus in both cell lines, which was maintained in Plasmax as well as in DBTRG-05MG cells in DMEM (Figure 9). So, 2-deoxyglucose can be explored in the future as an enhancer of the oncolytic activity of poliovirus in vivo.

### 2.9. Poliovirus Infection Does Not Trigger Oxidative Stress

**Figure 10:**
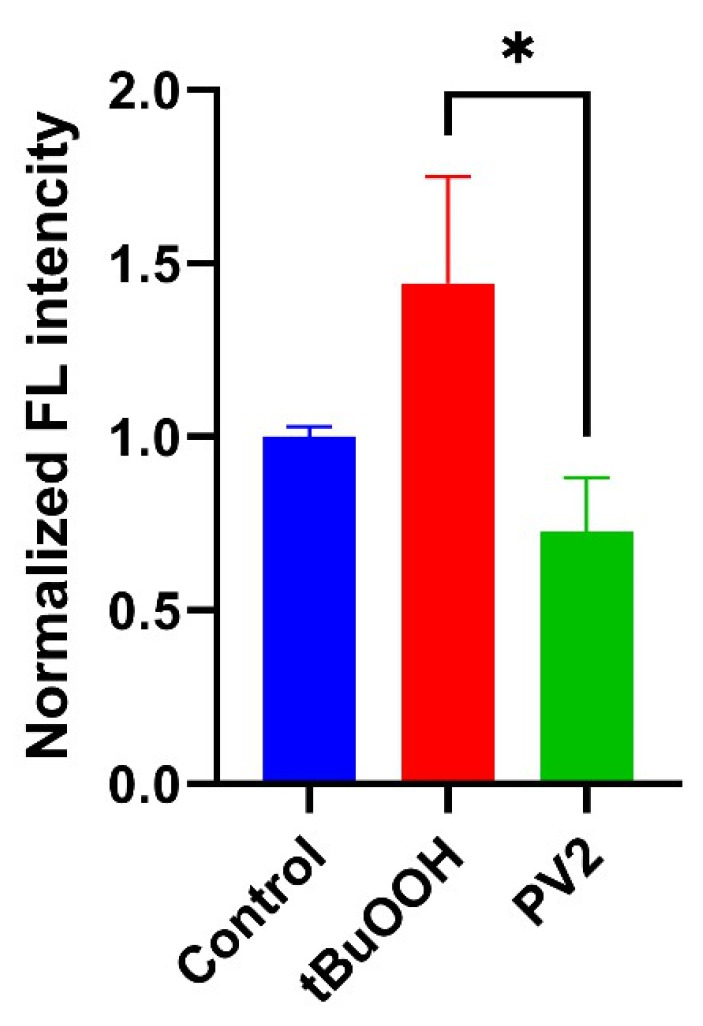
Poliovirus infection in U-251 MG cells is not accompanied by increased production of superoxide anion. Superoxide production was assessed using dihydroethidium staining with subsequent measurement of fluorescence of specific 2-hydroxyethidine product by flow cytometry. tBuOOH was used as positive control. Data are presented as mean ± SD. * *p* ≤ 0.05 by ANOVA with Tukey post hoc test.

**Figure 11:**
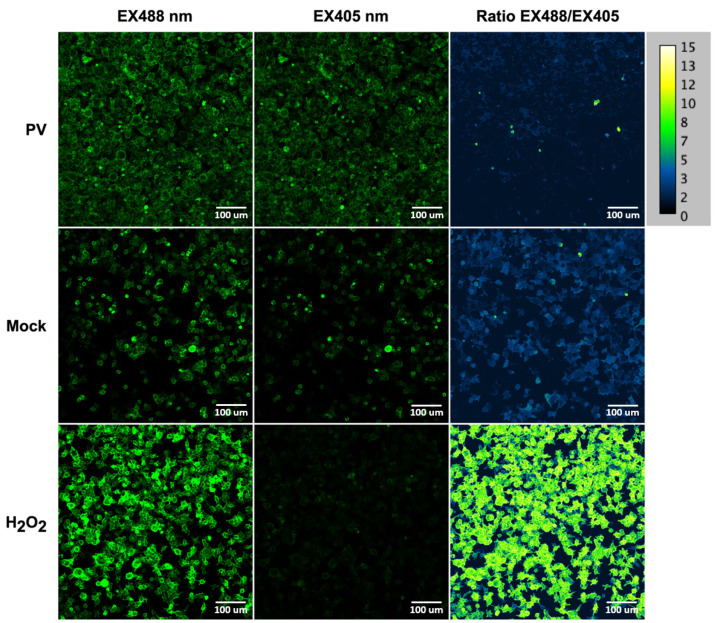
Poliovirus infection does induce changes in hydrogen peroxide levels. H_2_O_2_ levels in HEK293TΔIFNR1 cells stably expressing the genetically encoded ratiometric HyPer7-Lifeact sensor were measured by confocal microscopy. The cells were infected with poliovirus at MOI = 1 for 14 h; green fluorescence was quantified after excitation at 405 and 488 nm. As a positive control, the cells were treated with hydrogen peroxide 5 min prior to analysis. Ratiometric analysis is presented as the ratio of excitation wavelengths 488/405.

**Figure 12:**
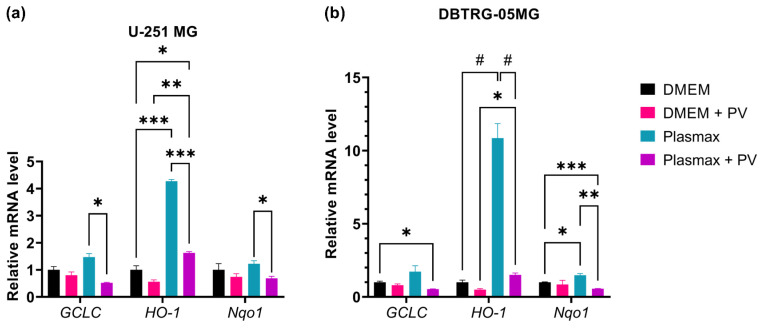
Relative levels of mRNA of Nrf2/ARE pathway in GBM cells. U-251 MG (**a**) or DBTRG-05MG (**b**) cells maintained in DMEM or Plasmax media were infected with PV at MOI 1, and mRNA levels were measured 24 h.p.i. by reverse transcription and real-time PCR analysis. mRNA levels were normalized to mRNA of β-glucuronidase and then to values of mock-infected cells in DMEM. Data are presented as mean ± SD, * *p* ≤ 0.05, ** *p* ≤ 0.01, *** *p* ≤ 0.001, ^#^ *p* < 0.07 by ANOVA with Tukey post hoc test.

Since various viruses trigger oxidative stress, our final goal was to reveal if poliovirus also affects the production of reactive oxygen species. Superoxide anion production was quantified by dihydroethidium (DHE) using FACS, while levels of hydrogen peroxide were assessed by the ratiometric genetically encoded HyPer7-LifeAct sensor and confocal microscopy. The results demonstrate that poliovirus replication does not affect the production of superoxide (Figure 10). Levels of H_2_O_2_ were also unaltered, as no changes in the HyPER7 fluorescence 488/405 ratio were registered in infected cells (Figure 11). Finally, we quantified the expression of genes regulated by the Nrf2 transcription factor, which is the master regulator of antioxidant defense [43]. We revealed no changes in their expression in both cell lines in DMEM, but revealed suppressed expression by poliovirus in cells maintained in Plasmax (Figure 12).

## 3. Discussion

Oncolytic viruses have a high potential in anticancer therapy [44]. Currently there are four viruses approved for clinical use. They include Talimogene laherparepvec (T-VEC, Imlygic) for the treatment of melanoma [45], Oncorine (H101) against head and neck cancer and esophagus cancer [46], Rigvir (ECHO-7) against melanoma [47], and Delytact (Teserpaturev/G47Δ) for the treatment of certain solid cancers, e.g., malignant glioma [48]. Many other oncolytic agents are currently in clinical trials (as exemplified by recent papers [49,50]). Anti-GBM potential is attributed to a wide array of natural or engineered viruses, including members of pox- [51], herpes [52], adeno- [53], paramyxo- [54], and flavi- [55,56] viruses. Our group expanded this list with enteroviruses, including coxsackieviruses and polioviruses [35,57]. Here we aimed to explore one of the options for enhancing the oncolytic activity of poliovirus against GBM.

It is well established that different viruses reprogram metabolic and redox pathways of host cells to ensure efficient replication and virion production [16,58,59]. Many of these changes are strongly associated with the pathogenesis of viruses. As an array of compounds that target metabolic enzymes are used as drugs for the treatment of cancer or autoimmune disease (such as metformin, methotrexate, sulfasalazine, mycophenolic acid, etc.) [60,61], their repurposing as enhancers of oncolysis is attractive. Indeed, we previously reported the enhancement of oncolytic activity of coxsakievirus type 5 by 2-deoxyglucose, which targets upstream stages of glycolysis [35]. However, such studies require understanding how a particular oncolytic virus depends on cell metabolism and interferes with it, as such drugs could interfere with the replication of the virus, thus decreasing the efficacy of virotherapy.

Investigation of cell metabolism is hampered by the imprint of standard culture media, such as DMEM and RPMI, on cell metabolism [36,62]. Therefore, one of the approaches in metabolomics is the usage of modern media compositions that resemble human plasma (i.e., HPLM and Plasmax [63,64]). Emerging reports show that the choice of culture media may affect the efficacy of drugs [65,66,67]. So, here we analyzed the imprint of poliovirus on cell metabolism in classical (DMEM) and physiological (Plasmax) media. We showed that the choice of culture media affects rates of poliovirus infection: Plasmax suppressed virus replication in U-251 MG cells but was upregulated in DBTRG-05MG cells. Together with our previous data on the absence of the Plasmax effect on PV replication in RD cells [37], the effect of media on the replication of viruses is cell line-specific. Moreover, this report is the first example of enhanced replication of viruses in Plasmax, as many other infections, including hepatitis C virus, SARS-CoV-2, and the influenza virus, had significantly lower rates of infection in Plasmax [37]. We also revealed a correlation between the efficiency of poliovirus replication and mitochondria morphology: a high rate of replication required fragmentation of the mitochondrial network. Furthermore, in these cells with fragmented mitochondria, the virus suppressed glycolysis. This could be required for ensuring a high cytopathic effect of the virus, as 2-deoxyglucose, which inhibits the early steps of glycolysis, significantly potentiated cell death. This leads to two conclusions. First, 2-deoxyglucose can indeed be investigated as a drug to potentiate enterovirus-induced oncolysis, as previously published for coxsackievirus [35]. Second, the role of mitochondria in the replication of members of the *Picornaviridae* family merits further study. Specifically, it is worth investigating the role of mitochondria-associated membranes (MAMs—tight contacts between mitochondria and the endoplasmic reticulum), as such contacts were already shown to be critical for replication of the hepatitis C virus [68]. MAMs were also shown to be disrupted by dengue and Zika viruses to ensure their high cytopathic effect [69]. In addition, these RNA viruses induce elongation of mitochondria, which is associated with the formation of membrane organelles for virus replication and downregulation of the innate immune response [70,71].

Our study also revealed that the imprint of poliovirus infection on cell metabolism is cell line-dependent and is affected by the culture medium. An increase in levels of amino acids and nucleic bases/nucleosides may indicate reprogramming of cell metabolism to enhanced synthesis of building blocks that are essential for virion production. It is a well-known fact that the addition of glutamine and glucose to the medium of cultured cells enhances assembly and the release of viral particles [72]. Accumulation of intermediates of glycolysis can indicate the suppression of this pathway. Indeed, the Seahorse assay revealed that poliovirus downregulates glycolysis as well as mitochondrial respiration. It is tempting to assume that the suppression of both these ATP-producing pathways contributes to the cytopathic effect of the virus. At the same time, metformin and phenformin, which inhibit respiratory complex I [73,74], did not affect the virus replication/cytopathic effect, with the exception of metformin in the case of U-251 MG cells. So, it can be concluded that respiration is dispensable for poliovirus.

We showed that poliovirus suppressed the basal respiratory activity and spare respiratory capacity of U-251 MG cells in both media but not in DBTRG-05MG cells. Simultaneous reduction in both activities suggests that it was more likely due to decreased mitochondria biogenesis or more rapid turnover, rather than to decreased concentrations of substrates for the electron transport chain and TCA cycle activity [75]. At the same time, we did not reveal any noticeable differences in mitochondria staining intensity while analyzing their morphology. So, the differences could also be mediated by more complex mechanisms, such as the assembly of respiratory supercomplexes [76,77] or the altered interaction of these organelles with lipid droplets [78] or the endoplasmic reticulum, as already mentioned above [79].

A pronounced accumulation of DHAP, shown here in U-251 MG cells, can be a marker of decreased cell viability [80,81,82], rather than a marker of increased biosynthesis of triglycerides. Indeed, we failed to find a correlation between the accumulation of DHAP and neutral lipids. Temporary accumulation of neutral lipids correlates with the previously published data [41].

We also revealed that poliovirus interferes with polyamine metabolism in a cell line-specific manner. Biogenic polyamines are positively charged aliphatic molecules present in all types of human cells in millimolar and submillimolar concentrations that are critical for their growth and differentiation [83,84,85,86]. Their levels are increased in tumors, making drugs that suppress their biosynthesis or enhance catabolism promising anticancer drugs [87]. Indeed, DFMO (Eflornithine^®^) demonstrated very promising results in clinical trials of the treatment of neuroblastoma, including relapsed cases [88,89,90], leading to its approval by the FDA under the Iwilfin trademark [91]. As polyamines have been discussed in the context of glioblastoma [92,93], DFMO has also been investigated as a component of anti-GBM therapy [94,95] and the treatment of human African trypanosomiasis [96]. So, it is not surprising that DFMO and other regulators of polyamine metabolism have been evaluated as antiviral agents. To date, DFMO has demonstrated activity against filoviruses [97], herpesviruses [98,99], alphaviruses [100,101], coronaviruses [26,102], flaviviruses [20,102], bunyaviruses [102], rhabdoviruses [102], hepadnaviruses [103], and picornaviruses [102], including coxsakievirus B3 and poliovirus [102]. The activity against the latter was shown by Mounce et al. in Vero E6 cells [102]. It correlates with a moderate reduction in PV replication in U-251 MG cells shown here. However, we did not reveal an antiviral effect for DENSpm, MDL72.527, SAM486a, or GC7, which have previously been described as antiviral agents against coxsackievirus, Zika and Chikungunya virus, as well as hepatitis C virus [20,27,97,102,104,105]. So, poliovirus is less dependent on polyamine metabolism than other infections, at least in GBM cells. Alternatively, glioblastoma cells could exhibit different polyamine metabolism compared to other tumor cell lines. This may result in a lower input of polyamine biosynthesis vs. import in maintaining the spermine and spermidine pool. Yet this assumption has never been verified; we previously reported that GBM cell lines differ from other cancer cell lines by the pronounced expression of PAOX, one of the polyamine-catabolizing enzymes [106].

The exact function of polyamines in the PV life cycle remains unknown. In the case of other infections, polyamines were shown to be incorporated into virions to support their infectivity (shown for bunyaviruses and herpes simplex virus [40,107]) or prevent the formation of defective virions (described for bunyaviruses [27]). They also support cholesterol metabolism and promote its incorporation into viral particles (shown for Rift Valley Fever virus and coxsackievirus [108,109]), and confer permissiveness of cells to viral infections (shown for coxsackievirus [110]). However, the cell background and mechanism of entry of a particular virus may affect its sensitivity to polyamine depletion.

The impact of viruses on polyamine metabolism has been studied less extensively than the role of polyamines in their replication. A reduction in polyamine levels has been shown for hepatitis C virus [20], chronic coxsackievirus infection [22], Kaposi’s sarcoma-associated virus [98], and SARS-CoV-2 [111]. Here we demonstrate a similar reduction in spermine in GBM cell lines that were cultured in a classical DMEM medium. It is noteworthy that spermine is the major polyamine in these cells when DMEM was used, whereas the replacement of DMEM with Plasmax leads to a substantial rise in the levels of spermidine. This indicates that the culture medium also affects polyamine metabolism. This is important for studies outside the virology field, as spermidine and its ratio to spermine are critical for the control of cell differentiation (e.g., [112]).

Finally, we revealed no indication of oxidative stress in GBM cell lines during poliovirus infection. This is contrary to the effect of human immunodeficiency virus (HIV) [113], hepatitis B and C viruses [29], herpes viruses [114], and a variety of acute respiratory infections [31], including SARS-CoV-2 [115]. Oxidative stress has also been described for enteroviruses, including Enterovirus 71 [116,117] and coxsackievirus [118]. However, increased ROS production during coxsackievirus infection, observed in vivo, could have resulted from indirect events, such as an inflammatory response.

Finally, the accumulation of such metabolites as succinate, fumarate, and 2-hydroxyglutarate (referred to as oncometabolites) is associated with the development of certain types of cancer. These metabolites act as signaling molecules, activating prooncogenic signaling pathways, promoting epigenetic changes, and conferring resistance to apoptosis [119]. This is especially important for gliomas, as many of these tumors have mutations in isocitrate dehydrogenase (IDH) 1/2 genes, leading to the production of 2-hydroxyglutarate [6]. However, the cell lines used in our study did not bear IDH1/2 mutations. So, the investigation of the imprint of oncolytic viruses on cell metabolism should in the future be examined in the context of IDH1/2 mutations to explore different variants of host cells.

## 4. Materials and Methods

### 4.1. Materials

(Dimethylamino)naphthalene-1-sulfonyl chloride (dansyl chloride), 1,6-diaminohexane, 1,7-diaminoheptane, O-methylhydroxylamine, and 4′,6-diamidino-2-phenylindole (DAPI) were purchased from Sigma-Aldrich (Darmstadt, Germany). MSTFA (N-methyl-N-(trimethylsilyl)trifluoroacetamide) was purchased from MedChemExpress (Monmouth Junction, NJ, USA). BODIPY 493/503 dye (4,4-difluoro-1,3,5,7,8-pentamethyl-4-bora-3a,4a-diaza-s-indacene) was from ThermoFisher Scientific (Logan, MA, USA). The metabolic inhibitors used and their vendors are listed in Table 1. All oligonucleotides were synthesized by Evrogen (Moscow, Russia).

### 4.2. Cell Cultivation

The DBTRG-05MG (CRL-2020) and HEK293T (CRL-3216) cell lines were obtained from the American Tissue Culture Collection (ATCC, Manassas, VA, USA). Human glioblastoma U-251 MG cells were obtained from the collection of the laboratory of cell proliferation, EIMB RAS. Authentication of these cell lines was confirmed by STR analysis (“Gordiz” company, Moscow, Russia). The HEK293TΔIFNR1 cell line was described earlier [120]. All cultures were routinely checked for mycoplasma contamination using the MycoReport test (Evrogen, Moscow, Russia).

The cells, cryopreserved in fetal bovine serum (FBS, Biosera, Cholet, France) with 10% DMSO, were seeded and maintained in DMEM (ServiceBio, Wuhan, China) supplemented with 10% FBS, 2 mM glutamine, 50 U/mL penicillin, and 50 µg/mL streptomycin at 37 °C in a humid atmosphere with 5% CO_2_. They were split every 2–3 days using 0.05% trypsin-EDTA solution (PanEco, Moscow, Russia). Alternatively, U-251 MG and DBTRG-5MG cells were maintained in a Plasmax medium [63] under similar conditions, with cultivation for at least 6 days in this medium prior to experiments.

### 4.3. Virus

The Sabin vaccine strain of poliovirus type 2 (PV), obtained from the collection of the laboratory of cell proliferation, EIMB RAS, was used in this work. The virus was propagated in HEK293T with subsequent production of viral stock with a titer of 10^9^ virus particles/mL.

### 4.4. Cell Morphology Analysis

Twenty-four hours prior to infection with the virus, cells were seeded on 6 cm Petri dishes at 1.5 × 10^6^ cells/well density, 6-well plates at 0.5 × 10^6^ cells/well density, or on 96-well plates at 2 × 10^4^ cells/well density. Morphology before and after infection was visualized on a ZOE fluorescence microscope (BioRad, Hercules, CA, USA) in brightfield mode.

### 4.5. Replication Assay

To determine the rates of virus replication, cells were infected at MOI = 1, 3, and 10, and the virus was then collected from supernatants or cryolyzed cells at 2, 6, 12, and 24 h post-infection. Virus titers were estimated by infecting HEK293T cells with serial dilutions of supernatants according to Reed and Muench [121]. Titration was estimated to be 72 h.p.i. by visual determination of CPE using microscopy.

### 4.6. RT-qPCR

The cells were seeded on 6 cm Petri dishes, infected with the virus in an FBS-free medium at MOI = 1. Twenty-four hours later, the cells were harvested, and RNA was isolated using the RNA extraction kit (Biolabmix, Novosibirsk, Russia) and subjected to recombinant DNAse (Roche, Basel, Switzerland) treatment. Reverse transcription was carried out using a random hexamer primer and the RevertaL enzyme (Amplisence, Moscow, Russia). PCR was performed using primers listed in Table 2. The standard reaction (10 μL) contained respective primers (0.8 µM each), cDNA equivalent to 10 ng of total RNA, and qPCRmix-HS SYBR (Evrogen, Moscow, Russia). The PCR thermal conditions for initial DNA denaturation were 55 °C for 5 min and 95 °C for 10 min, followed by 40 amplification cycles each of 95 °C for 10 s and 57 °C for 1 min. Afterwards, DNA melting was performed to confirm amplification specificity. The results of RNA-qPCR were analyzed using the ΔΔCt approach.

### 4.7. Cell Viability Assays

Cells were seeded in 96-well plates (TPP, Trasadingen, Switzerland) at a density of 2 × 10^4^ cells/well. Twenty-four hours later, compounds were added, followed by the addition of poliovirus at multiplicities of infection (MOI) 0.00005–50 with 10-fold serial dilutions. As the control, untreated cells were infected with the virus at the same MOI. Cell viability was assessed twenty-four hours post-infection (unless otherwise stated) by treatment with 44 µM resazurin for 3 h and the measurement of fluorescence at 600 nm with excitation at 545 nm using a ClarioStar Plus microplate reader (BMG LabTech, Ortenberg, Germany).

### 4.8. Measurement of Glycolysis and Mitochondrial Respiration

Glycolysis and mitochondrial respiration were assessed by Seahorse technology on an XFe24 analyzer (Agilent Technologies, Santa-Clara, CA, USA) according to the manufacturer’s instructions with slight modifications. Briefly, 24 h prior to analysis, the cells were seeded via XF24 Cell Culture Microplate into (1.5 × 10^5^ cells/well) in DMEM or Plasmax media with four replicates. For the MitoStress test, 45 min before analysis the media was changed to DMEM or Plasmax lacking phenol red dye and bicarbonate and supplemented with 25 mM (DMEM) or 5.5 mM (Plasmax) glucose, 2 mM pyruvate, and 2 mM glutamine, and the plate was kept at 37 °C in a normal atmosphere. To evaluate respiration-linked ATP production, maximum respiratory capacity, and non-mitochondrial respiration, ATP-synthase inhibitor oligomycin, uncoupler FCCP, and a mixture of complex I and III inhibitors rotenone and antimycin were added to final concentrations of 1 µM (oligomycin), 0.45 µM (FCCP), and 1 µM each (rotenone/antimycin). For each condition, three readings were performed at 3 min intervals.

In the case of the GlycoStress test, 30 min prior to analysis, the medium was changed to DMEM or Plasmax lacking phenol red dye, bicarbonate, and glucose and supplemented with 2 mM pyruvate and 2 mM glutamine. During the assay 5.5 and 25 mM glycose, 1 µM oligomycin, and 50 mM 2-deoxyglucose were added to assess basal glycolysis, maximal glycolytic capacity, and non-glycolytic acidification, respectively. The raw data were processed by Seahorse Wave Desktop software v.2.6.1.53 (Agilent Technologies, Santa-Clara, CA, USA).

### 4.9. Immunostaining

Cells were seeded in 48-well plates (TPP, Trasadingen, Switzerland) at a density of 1.2 × 10^5^ cells/well. Twenty-four hours later, poliovirus was added at MOI 10, 3, 1, or 0.1. After 24 or 48 h, the medium was removed and the cells were washed with phosphate buffer saline (PBS). Next, the cells were fixed with paraformaldehyde (PFA) at room temperature, permeabilized with 0.5% Triton X-100, washed with PBS, and 3% BSA in PBS was added; the cells were incubated for 1 h at 37 °C. Primary mouse antibodies to poliovirus (Santa Crus Biotechnologies, Santa-Clara, CA, USA, #sc-80633) were diluted in 2% bovine serum albumin (BSA) in PBS, added to the wells, and incubated for 1 h at 37 °C. Then the cells were washed with PBS and incubated with FITC-conjugated secondary goat anti-mouse IgG (cat # S0007, Affinity Biosciences, Cincinnati, OH, USA) in PBS containing 1% BSA for 1 h at 37 °C. After the washing step, DAPI was added to the cells to a 300 nM final concentration for 5 min at RT. The results were visualized on a ZOE fluorescence microscope (BioRad, Hercules, CA, USA) in blue and green channels and processed in ImageJ v.1.54g (NIH, Bethesda, MD, USA).

### 4.10. Liquid Chromatography

The cells were grown on 6-well plates. After removal of the culture cell medium, cells were washed twice with 1 mL PBS on ice. Then 0.1 mL mQ was added per well, and the plate was subjected to three cycles of freezing (−196 °C) and thawing at +4 °C. After the final thaw, samples were transferred to 0.5 mL polyethylene tubes. Each well was washed with an additional 0.1 mL mQ, combined with the crude cell lysates, and subjected to ultrasound treatment for 3 min at 0–4 °C. Debris was removed by centrifugation for 10 min at 14,000× *g*. Total protein content in supernatants was measured using the Pierce™ BCA Protein Assay Kit (Thermo Scientific, Waltham, MA, USA) prior to precipitation of protein by the addition of 60% perchloric acid to the final 3% concentration with subsequent clarification by centrifugation for 10 min at 14,000× *g*. The clarified lysates were lyophilized and kept at −80 °C prior to analysis.

Polyamines were quantified by high-pressure liquid chromatography (HPLC) with precolumn derivatization with dansyl chloride. The analysis was performed by dissolving dry samples in 100 µL of saturated at 20 °C Na_2_CO_3_ aqueous solution. A different ratio mixture of 1,6-diaminohexane and 1,7-diaminoheptane was used as an internal standard, and hydrazine hydrate (2 µL) was applied to quench the dansylation reaction.

The solution of the dansylated polyamines in toluene (after two-step extraction with 200 µL of toluene) was vacuum dried; the residue was dissolved in 200 µL of methanol and applied on a reversed-phase column (Cosmosil C18-MS-II, 250 × 4.6 mm, 5 µm, 100 Å). The column was eluted (1 mL/min) with the following gradient: 0 min—0% B; 5 min—0% B; 60 min—100% B; 65 min—100% B; 70 min—0% B; and 75 min—0% B. System A—30% acetonitrile, 69.5% H_2_O, and 0.5% propionic acid. System B—79.5% acetonitrile, 20% tetrahydrofuran, and 0.5% propionic acid. Column temperature 40 °C, pressure 80–120 bar, fluorescent detection: λ_ex_ 340 nm and λ_em_ 530 nm (detector RF-20A, Shimadzu Scientific Instrument, Columbia, MD, USA).

### 4.11. Metabolite Quantification by Gas Chromatography-Mass Spectrometry

Metabolite quantification was performed by the GC-MS method. The cells were grown on 6 cm dishes, infected with the virus at MOI 1, harvested using a trypsin-EDTA solution, and subjected to subsequent centrifugation at 3600 rpm. After washing with PBS, each sample was divided into 1/10 for the quantification of total protein and 9/10 for metabolite analysis. The pellets were stored at −80 °C prior to further processing. Total protein was quantified with the Micro BCA kit (Thermo Fischer Scientific, Waltham, MA, USA).

Metabolites were extracted according to Fiehn’s protocol [122]. Each pellet was resuspended by vortexing in 1 mL of a chilled mixture of acetonitrile–2-propanol–water (3:3:2), supplemented with 3.4 µmol nor-valine, shacked for 20 min at 4 °C, vacuum-dried, dissolved in a mixture of acetonitrile–water, and again dried. Metabolites were derivatized with O-methylhydroxylamine, shacked for 1.5 h at 30 °C, and later with MSTFA with 30 min shacking at 37 °C as described in [123]. Measurements were carried out on a gas chromatography-mass spectrometer coupled with a monoquadrupole mass spectrometric detector (Crystal 5000, Yoshkar-Ola, Russia). Samples (1 μL) were injected into a helium stream at a rate of 1 mL/min; electron ionization was performed at 20 meV. Metabolites were separated on an HP-5 ms column (30 mm × 0.25 mm × 0.25 μm) (Agilent Technologies, Santa Clara, CA, USA). The injector, transfer line, and ion source temperatures were set to 250 °C, 290 °C, and 230 °C, respectively. Raw spectra were analyzed by Chromatec Analytic Software v.3.0.0.2 (Yoshkar-Ola, Russia). Quantification of the compounds was performed in the single ion monitoring mode in each channel, determining for each metabolite a characteristic ion in a narrow range of retention times for a given compound. Retention times and masses were obtained by analyzing standards of the corresponding compounds, carried out using the same derivatization protocol, analytical parameters, and instruments, and are listed in Table S1. The concentration of each compound was calculated from the peak intensity using a calibration curve obtained under the same experimental conditions. The values were then normalized to the signal of nor-valine used as an internal standard, as well as to the corresponding values of these metabolites obtained from pooled control samples.

### 4.12. Interferon Cell Treatment

U-251 MG and DBTRG-05MG cells were treated with 1000 IU of interferon α-2b (Vector-Medica, Novosibirsk, Russia) and incubated under standard conditions in a cell incubator for 24 h for subsequent GC-MS analysis and PCR verification. For this purpose, a commercial IFNα-2b lyophilizate containing 3 × 10^6^ IU was diluted in 1 mL of PBS and aliquoted into 50 μL aliquots that were stored at −20 °C for no more than a month.

### 4.13. Staining of Neutral Lipids

Neutral lipids were quantified by staining using BODIPY 493/503 dye. Cells were plated in 12-well plates and infected with poliovirus at MOI 1 when subconfluency was achieved. After 24 h.p.i., the culture fluid was removed and the cells were washed with PBS, fixed in 4% PFA at room temperature for 20 min, and washed with PBS again. Then 250 μg/mL BODIPY 493/503 in DMSO was added, and the cells were incubated for 15 min in the dark. The dye-containing solution was removed, the cells were washed again with PBS, treated with a 300 nM DAPI solution for 5 min in a CO_2_ incubator, and washed again with PBS. Fluorescence was visualized using a ZOE fluorescent cell analyzer (BioRad, Hercules, CA, USA) and processed in ImageJ (NIH, Bethesda, MD, USA).

### 4.14. Flow Cytometry

Production of reactive oxygen species was assessed using superoxide-sensitive fluorescent dihydroethidium (DHE) dye. The cells were stained as described earlier [124]. The intensity of fluorescence corresponding to a specific product (2-hydroxyethidine) was measured by flow cytometry at 405/610 nm [124,125] on a BD LSR Fortessa Cytometer (Becton Dickinson, Franklin Lakes, NJ, USA).

### 4.15. Sensors/Confocal Microscopy

Hydrogen peroxide levels were assessed by a genetically encoded HyPer7 sensor fused to β-actin (HyPer7-LifeAct) [126]. Its gene was amplified from the plasmid pLifeAct-HyPer7 kindly provided by Prof. Belousov (Federal Center for Brain and Neurotechnologies, FMBA of Russia, Moscow, Russia), using oligonucleotides Hyp7Lifeact-Puro-F (5′-atgctagcgccaccatgggc-3′) and Hyp7Lifeact-Puro-R (5′-atgaattctcaatcgcagatgaagctaac-3′) and cloned into the EcoRI site of a lentiviral pL4Puro vector. The lentivirus was assembled by the transfection of the resulting pL4Puro-HyPer7-LifeAct plasmid mixed with pLP1, pLP2, and pVSV-G (Invitrogen, Carlsbad, CA, USA). The medium containing the lentiviral particles was harvested, filtered through a 0.22 µm filter, and used for transduction of HEK293TΔIFNR1 cells with subsequent selection using puromycin, similar to as described previously [127].

For H_2_O_2_ analysis, HEK293TΔIFNR1-HyPer7-Lifeact cells were seeded on 35 × 10 mm confocal Petri dishes with a glass insert (SPL LifeSciences, Pocheon-si, Republic of Korea). Subconfluent cells were infected with poliovirus at MOI 1 for 7 and 14 h. As a positive control, the cells were treated with 200 μM H_2_O_2_ 5 min prior to analysis. Microscopic imaging and ratiometric analysis were performed using a Nikon Ti2 Eclipse confocal microscope (Nikon Microscope Solutions, Amstelveen, North Holland, The Netherlands) equipped with a CFI Plan Apochromat Lambda D 10× objective lens. HyPER7 fluorescence was registered in the 499–563 nm range after excitation either with 405 nm or 488 nm. The ratiometric index was calculated as a ratio of λex488/λex405 fluorescence.

### 4.16. Evaluation of Mitochondrial Morphology by Confocal Microscopy

The morphology of mitochondria was analyzed by confocal microscopy, as described by us earlier [37].

### 4.17. Statistical Analysis

All data were obtained from at least three independent experiments. The data are presented as means ± the standard deviation (SD). Statistical significance was determined by two-tailed unpaired *t*-test (pairwise comparison) or by two-way Analysis of Variance (ANOVA), followed by Tukey’s or Bonferroni post hoc test using GraphPad Prism 9.5 (GraphPad Software, La Jolla, CA, USA). A *p*-value of ≤0.05 was considered significant.

## 5. Conclusions

To sum up, we have shown that poliovirus reprograms the metabolism of glioblastoma cells. However, the imprint is dependent on the cell line and culture medium used. Specifically, in a physiological Plasmax medium, poliovirus decreases levels of alanine, proline, and phenylalanine, and increases levels of spermine, histidine, leucine, and isoleucine. We have also revealed that efficient replication of poliovirus in glioma cells is associated with the fragmentation of mitochondria and suppression of glycolysis. Altogether, poliovirus was less dependent on the metabolic pathways of host cells than other RNA viruses. The exceptions are the biosynthesis of polyamines and the import of pyruvate into mitochondria. We also showed that this virus does not induce oxidative stress, in contrast to such viral infections as hepatitis C, SARS-CoV-2, or the influenza virus.

The most translationally important result of our study is the enhancement of the cytopathic activity of poliovirus towards glioblastoma cells by 2-deoxyglucose. It suggests that inhibitors of glycolysis can be repurposed to enhance the action of oncolysis. However, such activity of 2-deoxyglucose, as well as that of other glycolytic inhibitors, should be verified in vivo. From the point of view of basic molecular virology, our study points to the role of mitochondria in the replication of picornaviruses as one of the directions for future studies.

## Figures and Tables

**Table 1:** Metabolic inhibitors and pharmacological agents whose activity was tested in this work.

Compound	Target	Metabolic Pathway	Source
2-Deoxyglucose (2DG)	Inhibitor of glycolysis	Glycolysis	Sigma (St. Louis, MO, USA)
5.33756.0001	ACSS	Fatty acid biosynthesis	Sigma
APCHA	Spermine synthase (SMS)	Polyamine biosynthesis	Sigma
AR-C155858	MCT1 and MCT2 inhibitor	Glycolysis	MedChemExpress (Monmouth Junction, NJ, USA)
Deferiprone (DFP)	Hypusination (DOHH inhibitor)	eIF5a hypusination	Sigma
DFMO	Ornithine decarboxylase (ODC)	Polyamine biosynthesis	MedChemExpress
Diethylnorspermine (DENSpm)	Spermidine/spermine-N1-acetyltransferase (SSAT)	Inducer of polyamine catabolism	Santa-Cruz Biotechnologies (Dallas, TX, USA)
Etomoxir	Inhibitor of carnitine palmitoyltransferase-1 (CPT-1)	Fatty acid oxidation	Sigma
GC7	Hypusination (DOHH inhibitor)	eIF5a hypusination	Santa-Cruz Biotechnologies
MCHA	Spermidine synthase (SRM)	Polyamine biosynthesis	Sigma
MDL72.527	Polyamine oxidases (PAOX, SMOX)	Polyamine catabolism	Sigma
Metformin	Inhibitor of mitochondrial respiratory complex I	Mitochondrial respiration system	Sigma
Phenformin	Inhibitor of mitochondrial respiratory complex I	Mitochondrial respiration system	Sigma
Sardomozide (SAM-486a)	AdoMetDC (ADM1)	Polyamine biosynthesis	MedChemExpress
UK-5099 (PF-1005023)	Inhibitor of the mitochondrial pyruvate carrier (MPC)	Link between glycolysis and TCA cycle	Sigma

**Table 2:** Sequences of primers used in the research.

Name	5′–3′ Sequence	5′–3′ Sequence
AGPS	GCGCGAGCTACGGGTCTG	CTCTCCGCGCTTTGCACT
Aldo	GGCCCCACGATAGTGTGAAT	TCTCTAATGACCCCTGCCCT
CD155	CACTGTCACCAGCCTCTGG	GTTATCATAGCCAGAGATGGATACCTC
DGAT	TATTGCGGCCAATGTCTTTGC	CACTGGAGTGATAGACTCAACCA
FASN	TACAAGCTGCGTGCCGCTGA	ACCCTCGATGACGTGGACGGAT
GCLC	GGATTTGGAAATGGGCAATTG	CTCAGATATACTGCAGGCTTGGAA
HO1	CCAGCAACAAAGTGCAAGATTC	TCACATGGCATAAAGCCCTACAG
IFNβ	GCCGCATTGACCATGTATGAGA	GAGATCTTCAGTTTCGGAGGTAAC
IFN-λ1	CGCCTTGGAAGAGTCACTCA	GAAGCCTCAGGTCCCAATTC
IGS15	GCCGATCTTCTGGGTGATCTG	ATGGGCTGGGACCTGACG
MxA	GGTGGTCCCCAGTAATGTGG	CGTCAAGATTCCGATGGTCCT
Nqo1	CCGTGGATCCCTTGCAGAGA	AGGACCCTTCCGGAGTAAGA
OAS-1	ACAACCAGGTCAGCGTCAGAT	AGGTGGTAAAGGGTGGCTCC
ODC	TTGCGGATTGCCACTGATGATTCC	ATCAGAGATTGCCTGCACGAAGGT
TPI	AGCTCATCGGCACTCTGAAC	CCGGGCGAAGTCGATATAGG

## Data Availability

The raw data supporting the conclusions of this article will be made available by the authors upon request.

## Supplementary Materials

The following supporting information can be downloaded at https://www.mdpi.com/article/10.3390/ijms26157346/s1.
