# Supplementary material for: Metabolic Imprint of Poliovirus on Glioblastoma Cells and Its Role in Virus Replication and Cytopathic Activity
Source: Int J Mol Sci. 2025 Jul 30;26(15):7346. doi: 10.3390/ijms26157346 (PMC12346930; doi:10.3390/ijms26157346)
Supplement: Supplementary file 1 [file ijms-26-07346-s001.zip › ijms-3682247-supplementary.pdf]

Supplementary data

Metabolic Imprint of Poliovirus on Glioblastoma Cells and Its Role in Virus Replication and Cytopathic Activity

Martin A. Zenov, Dmitry V. Yanvarev, Olga N. Ivanova, Ekaterina A. Denisova, Mikhail V. Golikov, Artemy P. Fedulov, Roman I. Frykin, Viktoria A. Sarkisova, Dmitry A. Goldstein, Peter M. Chumakov, Anastasia V. Lipatova and Alexander V. Ivanov *

Engelhardt Institute of Molecular Biology, Russian Academy of Sciences, 119991 Moscow, Russia;
martin.zenov@yandex.ru (M.A.Z.); yanvarev@eimb.ru (D.V.Y.); olgaum@yandex.ru (O.N.I.); katadenissova@yandex.ru (E.A.D.); cool.mik3492594@yandex.ru (M.V.G.); fedulovtt@gmail.com (A.P.F.); romanfrykin@mail.ru (R.I.F.); alice-lyddell@yandex.ru (V.A.S.); dmitritrygold@gmail.com (D.A.G.);
chumakovpm@yahoo.com (P.M.C.); lipatovaanv@gmail.com (A.V.L.)
* Correspondence: aivanov@yandex.ru or aivanov@eimb.ru

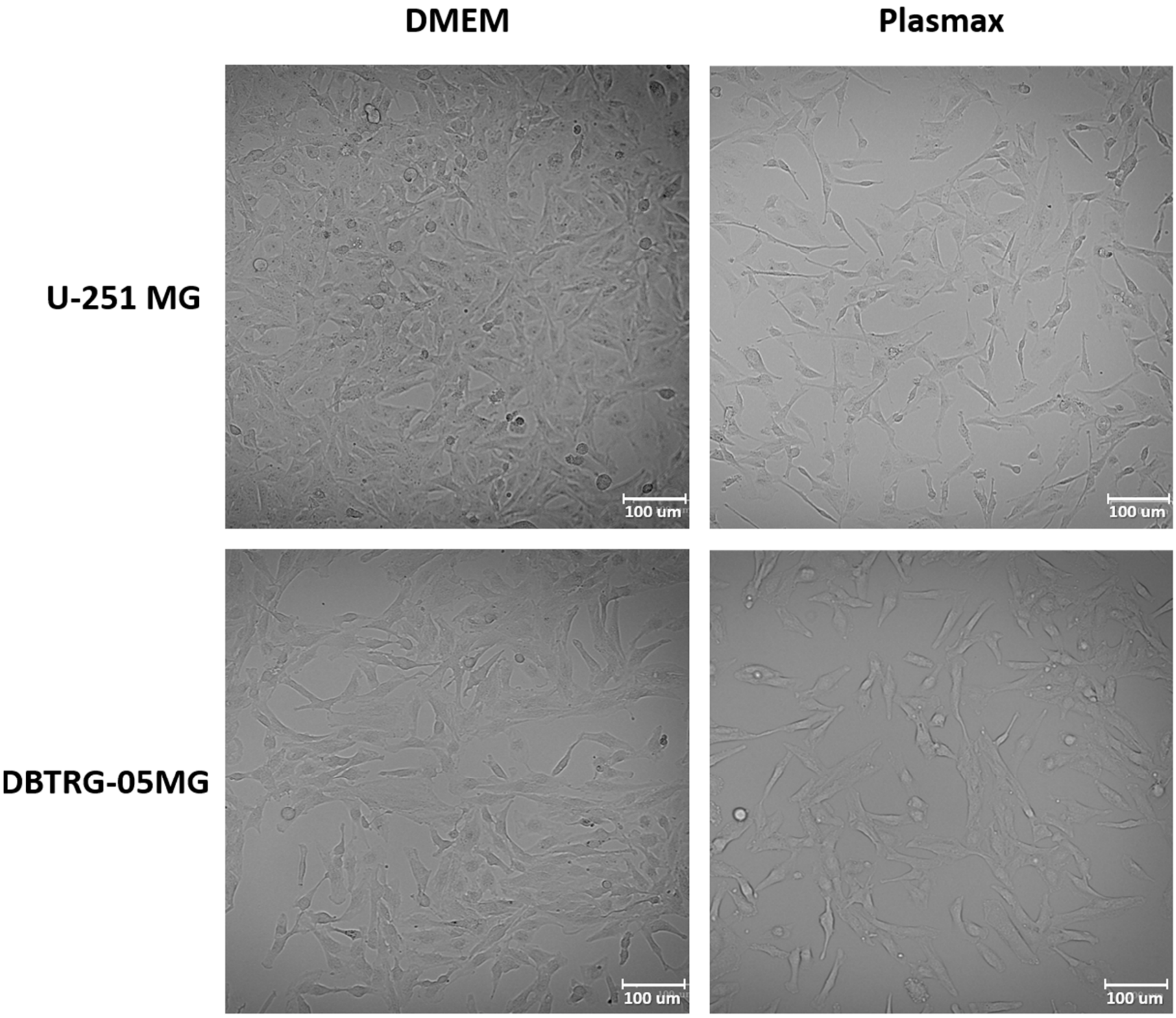

Figure S1. Morphology of GBM cell lines cultured in a conventional DMEM nutrient medium or in a plasma-like Plasmax medium.

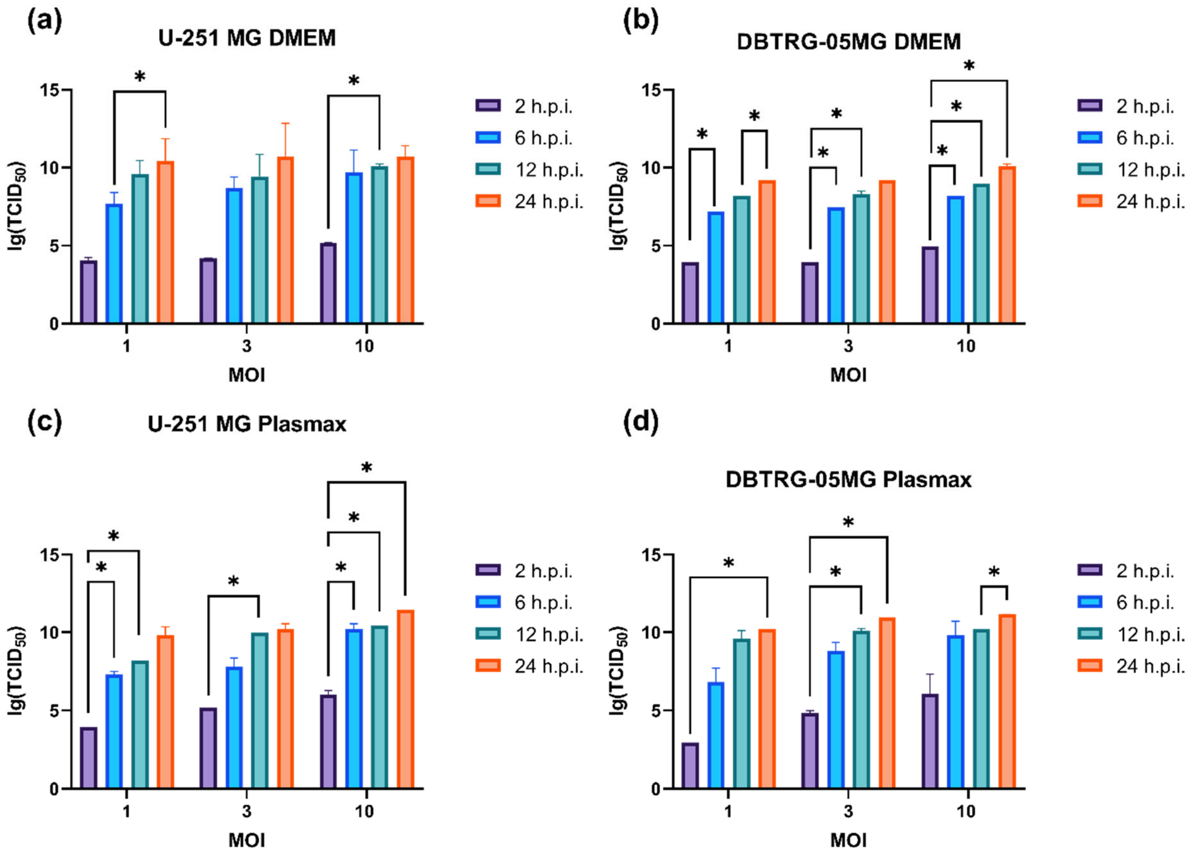

Figure S2. Evaluation of poliovirus replication kinetics in GBM cells. U-251 MG (**a**,**c**) or DBTRG-05MG (**b**,**d**) cells maintained in DMEM (**a**,**b**) or Plasmax (**c**,**d**) were infected with PV at MOI 1, 3, and 10, and $TCID_{50}$ was assessed 2, 6, 12, and 24 hours post-infection. The data are presented as mean ± SD, *$p \leq 0.05$ by two-way ANOVA with the Tukey post-hoc test.

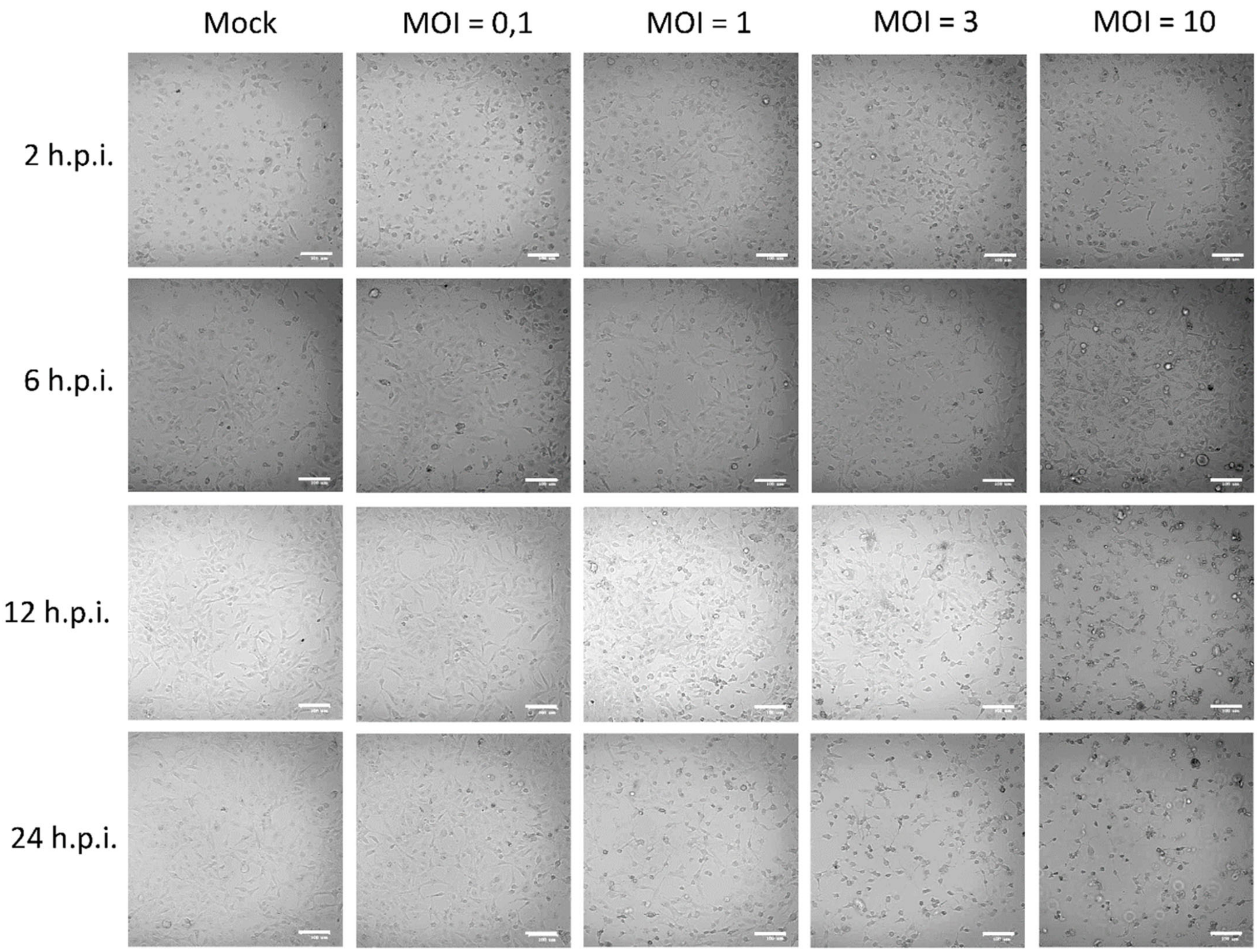

Figure S3. Cytopathic effect of poliovirus on U-251 MG cells. The cells maintained in DMEM were infected with poliovirus at various MOI, and cytopathogenic effect was visualized at 2-24 hours post-infection using Zoe system. The bar denotes 100 μm.

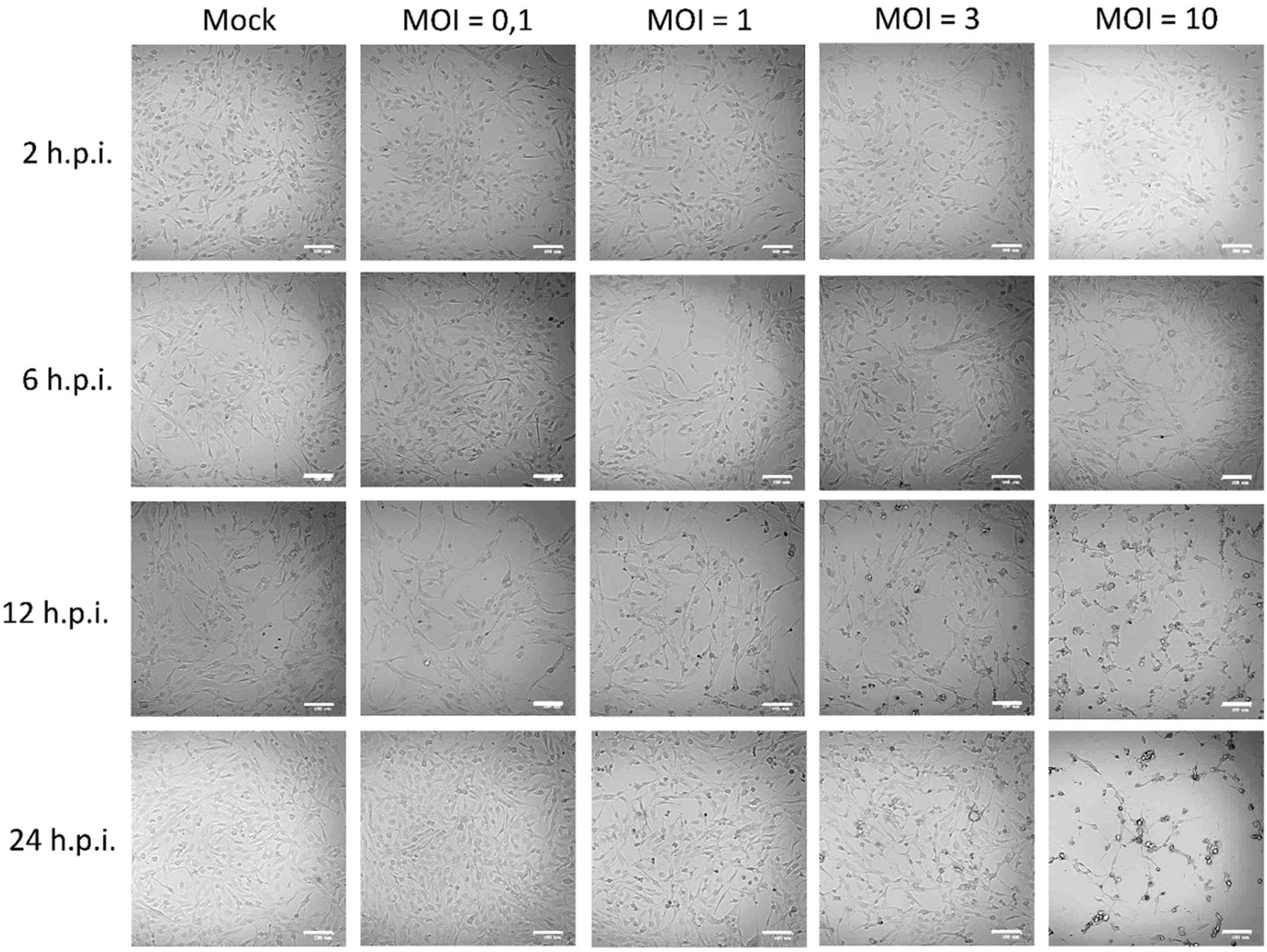

Figure S4. Cytopathic effect of poliovirus on DBTRG-05MG cells. The cells maintained in DMEM were infected with poliovirus at various MOI, and cytopathogenic effect was visualized at 2-24 hours post-infection using Zoe system. The bar denotes 100 μm.

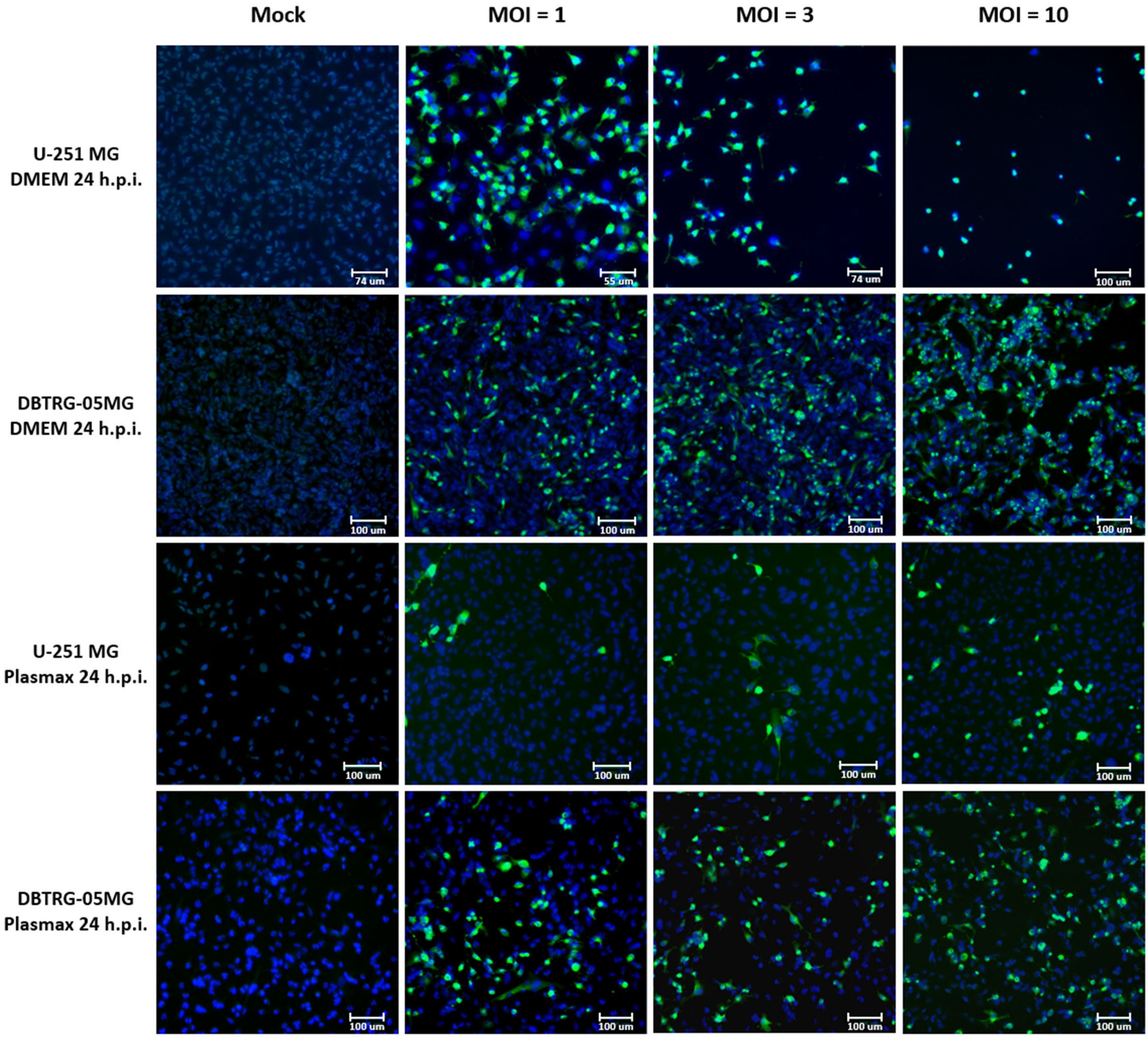

Figure S5. Levels of poliovirus infection in GBM cell lines at 24 hours post-infection (h.p.i.). U-251 MG or DBTRG-05MG cell lines maintained in DMEM or Plasmax media were infected with poliovirus at various MOI for 24 hours, and infection spread was monitored by immunostaining using primary antibodies to poliovirus and FITC-conjugated secondary antibodies.

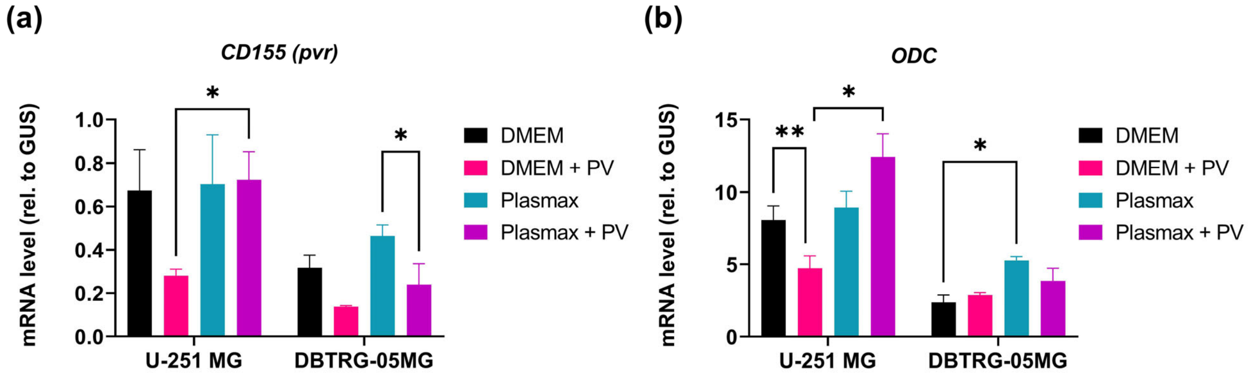

Figure S6. Levels of expression of CD155 (pvr) (**a**) and ornithine decarboxylase (ODC) (**b**) in GBM cell lines, measured by reverse transcription and real-time PCR analysis. The mRNA levels were normalized to mRNA of a β-glucuronidase. Data are presented as mean ± SD, $^*p \leq 0.05$ and $^{**}p \leq 0.01$ by ANOVA with the Tukey post-hoc test.

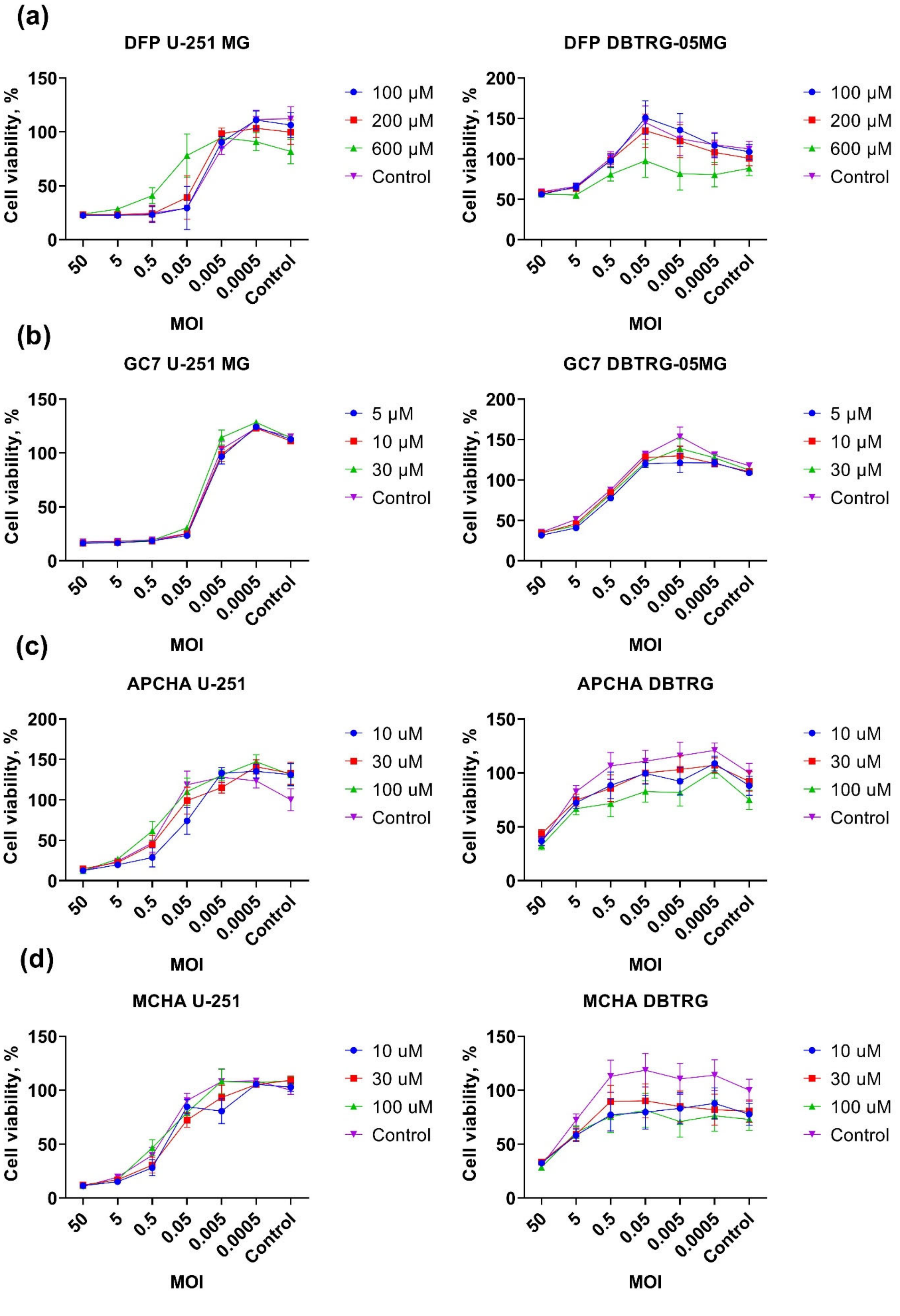

Figure S7. Cytopathogenic effect of poliovirus-infected GMB cells treated with inhibitors or inducers of polyamine-metabolizing enzymes. U-251 MG or DBTRG-05MG cells maintained in DMEM were pretreated with DFP (**a**), GC7 (**b**), APCHA (**c**) or MCHA (**d**),

and infected with PV at different MOI in the presence of these compounds at the same concentrations. Cell viability was measured using resazurin assay. The values were normalized to the values of untreated mock-infected cells. The data are presented as mean ± SD.

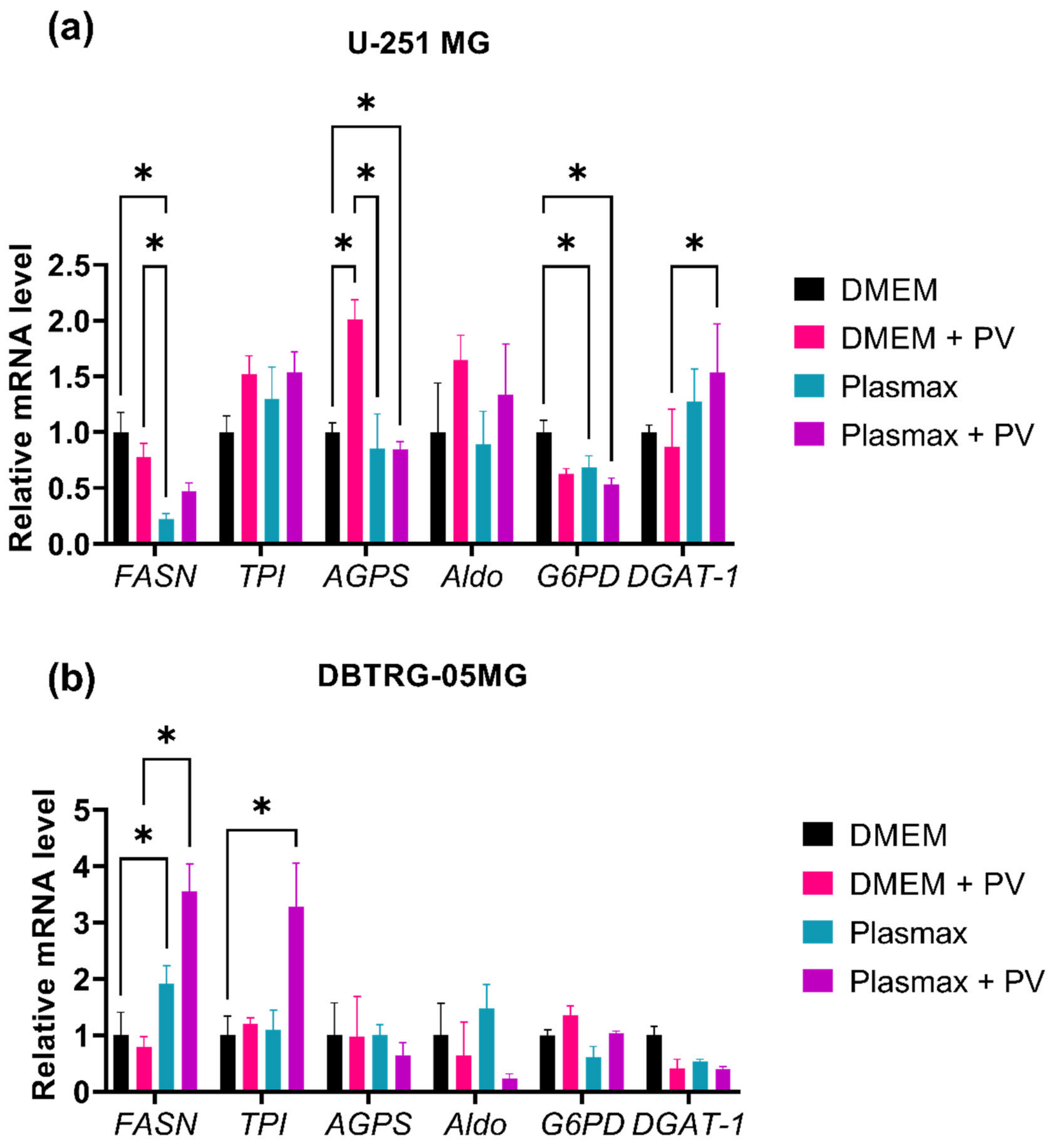

Figure S8. Relative mRNA levels of genes responsible for the regulation of glycolysis and triglyceride biosynthesis in GBM cells. U-251 MG (**a**) or DBTRG-05MG (**b**) cells maintained in DMEM or Plasmax media were infected with PV at MOI 1, and mRNA levels were measured 24 h.p.i. by reverse transcription and real-time PCR analysis. The mRNA levels were normalized to mRNA of a β-glucuronidase and then to the values of mock-infected cells in DMEM. Data are presented as mean ± SD, *$p \leq 0.05$ by ANOVA with the Tukey post-hoc test.

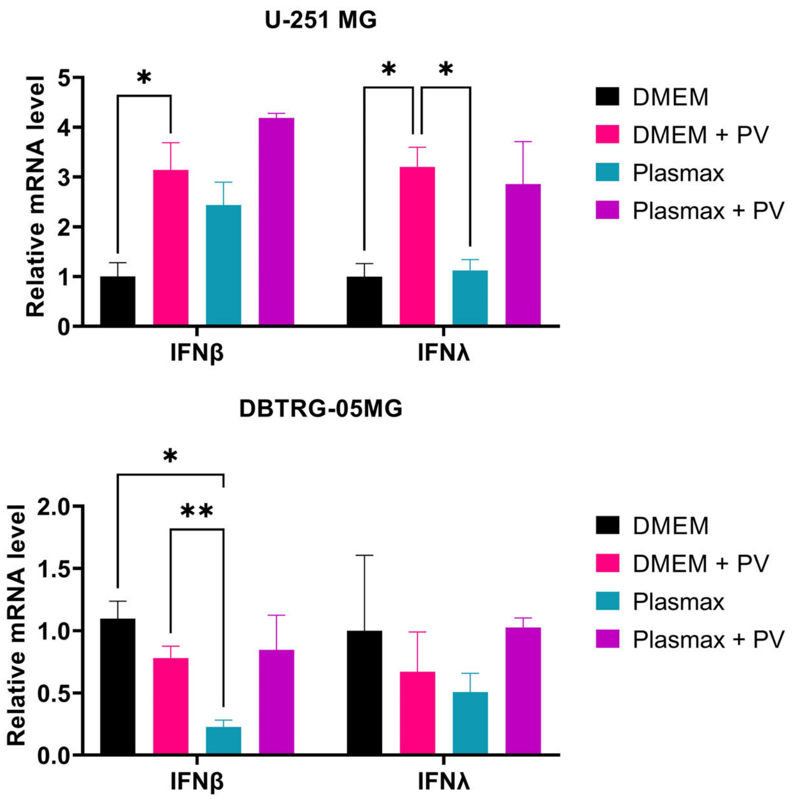

Figure S9. Poliovirus induces interferons β and λ in U-251 MG cells maintained in DMEM. Relative mRNA levels in U-251 MG or DBTRG-05MG cells were measured by reverse transcription and real-time PCR analysis. The mRNA levels were normalized to mRNA of a β-glucuronidase and then to the values of untreated cells in DMEM. Data are presented as mean ± SD, *$p \leq 0.05$ and **$p \leq 0.01$ by ANOVA with the Tukey post-hoc test.

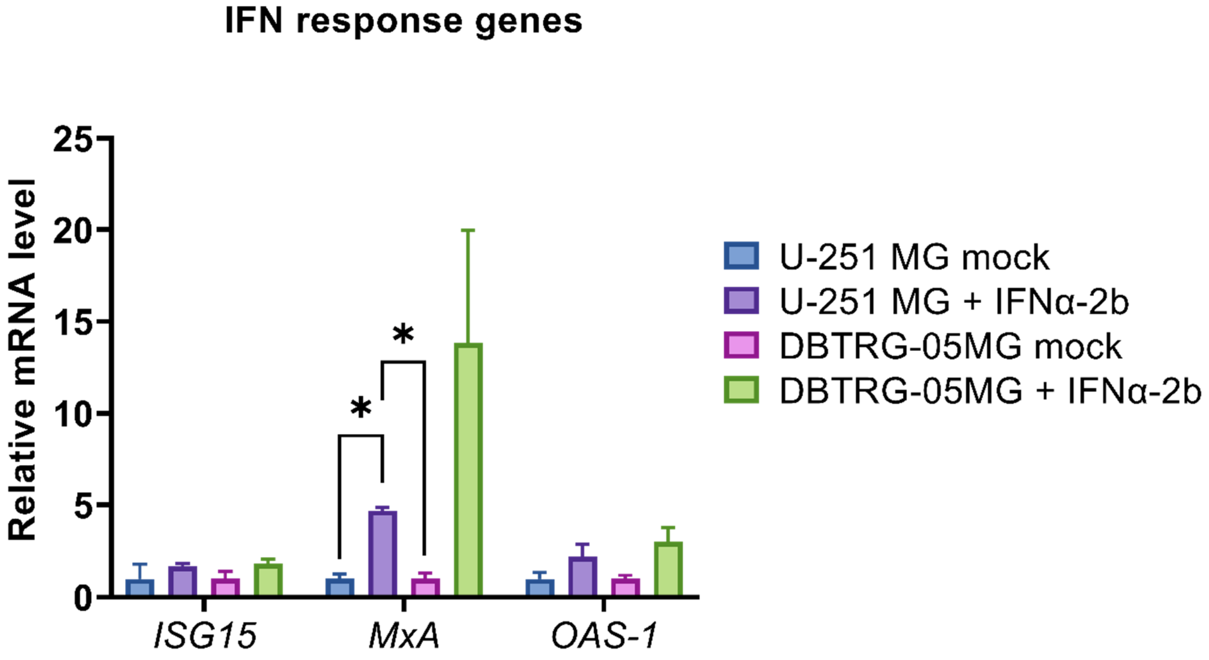

Figure S10. Verification of activity of recombinant interferon α-2b in GBM cell lines. Relative levels of mRNA of interferon-inducible genes in U-251 MG or DBTRG-05MG maintained in DMEM treated for 14 hours were measured by reverse transcription and real-time PCR analysis. The mRNA levels were normalized to mRNA of a β-glucuronidase and then to the values of untreated cells in DMEM. Data are presented as mean ± SD, *p ≤ 0.01 by ANOVA with the Tukey post-hoc test.

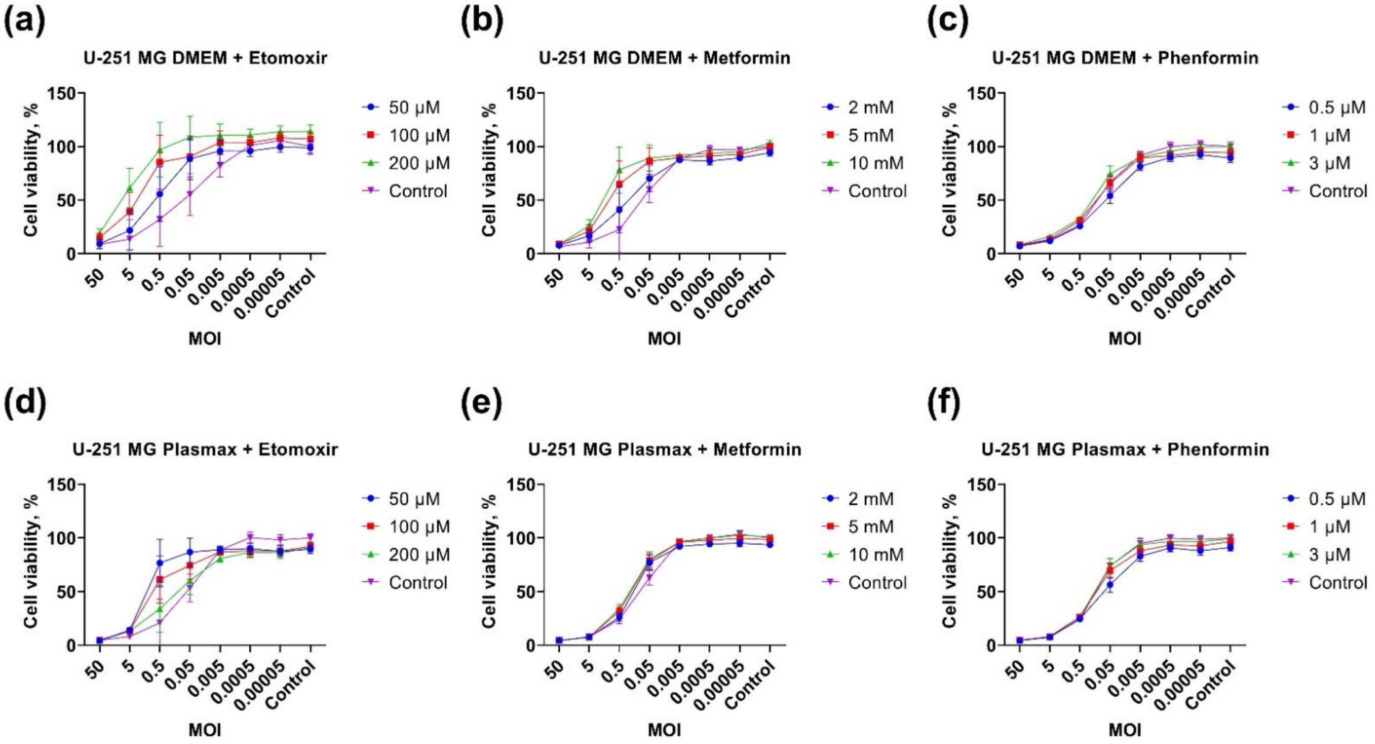

Figure S11. Cytopathogenic effect of poliovirus-infected GBM cells treated with inhibitors of metabolic enzymes. U-251 MG cells maintained in DMEM or Plasmax were

pretreated with etomoxir (**a**,**d**), metformin (**b**,**e**) or phenformin (**c**,**f**), and infected with PV at different MOI in the presence of these compounds at the same concentrations. Cell viability was measured using resazurin assay. The values were normalized to the values of untreated mock-infected cells. The data are presented as mean ± SD.

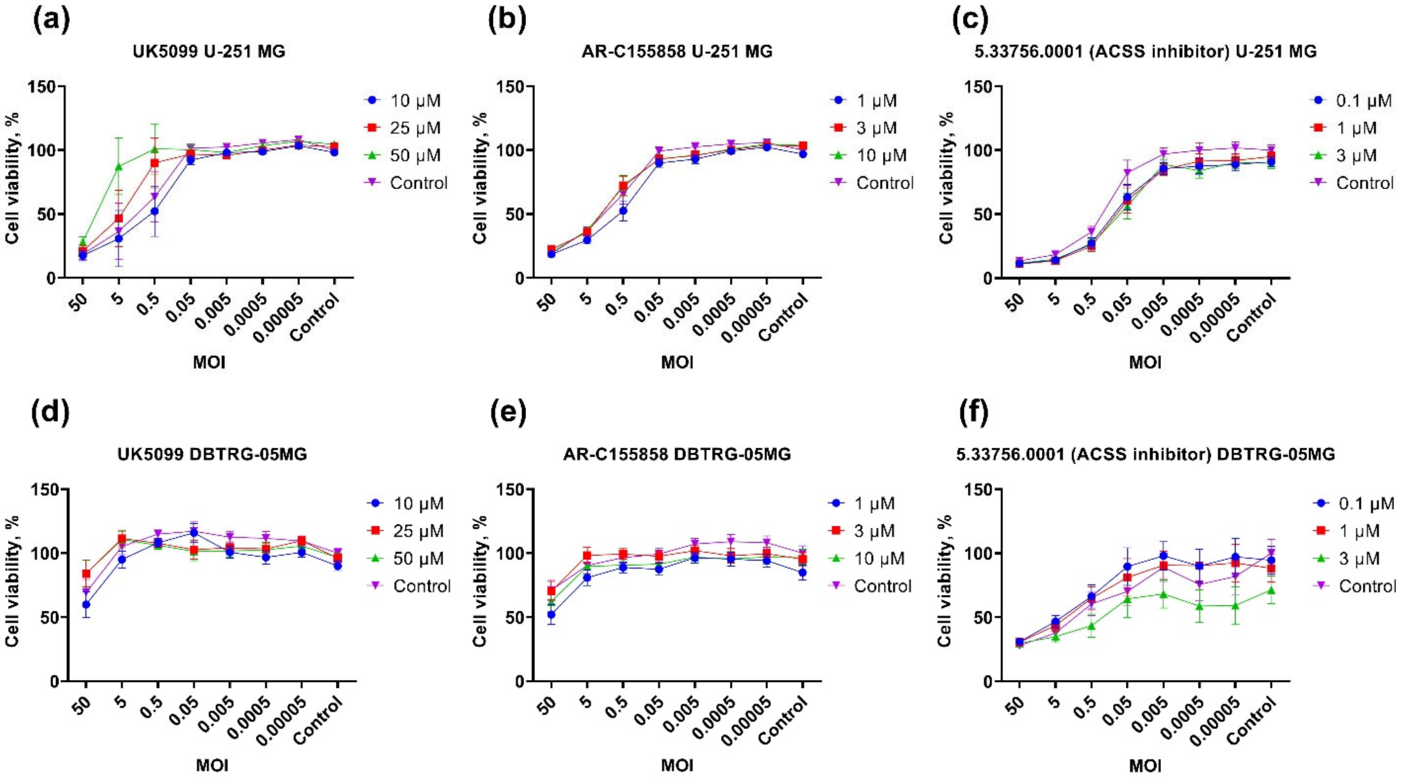

Figure S12. Cytopathogenic effect of poliovirus-infected GBM cells treated with inhibitors of metabolic enzymes or transporters. U-251 MG (**a**–**c**) or DBTRG-05MG (**d**–**f**) cells maintained in DMEM were pretreated with UK5099 (**a**,**d**), AR-C155858 (**b**,**e**), or 5.33756.0001 (**c**,**f**), and infected with PV at different MOI in the presence of these compounds at the same concentrations. Cell viability was measured using resazurin assay. The values were normalized to the values of untreated mock-infected cells. The data are presented as mean ± SD.

Table S1. Retention time and metabolite mass data used for gas chromatography-mass spectrometry.

Pathway	Full_name	Retention time (min)	m/z
Nucleot(s)ides	Adenine	15,54	264
Nucleot(s)ides	Adenosine	22,32	236
Amino acids	Alanine	6,11	116
Amino acids	Asparagine	13,35	116
Amino acids	Aspartic acid	11,58	232
TCA	Citrate	14,99	273
TCA	Cis-aconitate	14,23	229
Amino acids	Cysteine	12,01	220

Amino acids	Cystine	19,57	218
Nucleot(s)ides	Cytosine	11,57	240
Glycolysis	Dihydroxyacetone phosphate	14,16	315
Glycolysis	Fructose-1,6-bisphosphate	18,29	315
Glycolysis	Fructose-6-phosphate	19,62	315
TCA	Fumarate	9,38	245
Glycolysis	Glucose	15,86	205
Glycolysis	Glucose-1-phosphate	23,32	261
Glycolysis	Glucose-6-phosphate	19,73	387
Amino acids	Glutamic acid	12,77	246
Amino acids	Glutamine	14,51	156
Amino acids	Glycine	8,81	174
Nucleot(s)ides	Guanine	18,06	352
Nucleot(s)ides	Guanosine	23,31	324
Amino acids	Histidine	16,06	154
Amino acids	Homocysteine	13,30	234
Amino acids	Homocystine	21,49	278
Nucleot(s)ides	Hypoxanthine	14,88	265
Nucleot(s)ides	Inosine	21,83	230
Amino acids	Isoleucine	8,62	158
TCA	Itaconate	9,27	147
Glycolysis	Lactate	5,61	147
Amino acids	Leucine	8,34	158
Amino acids	Lysine	16,08	174
TCA	Malate	11,17	233
Amino acids	Methionine	11,57	176
Nucleot(s)ides	Orotate	14,19	258
TCA	Oxaloacetate	15,13	133
Amino acids	Phenylalanine	12,88	218
Glycolysis	Phosphoenolpyruvate	12,52	299
Amino acids	Proline	8,70	142
Amino acids	Pyroglutamic acid	11,61	156
Glycolysis	Pyruvate	5,49	174
Amino acids	Serine	9,50	218
TCA	Succinate	8,90	147
Amino acids	Threonine	9,84	117
Nucleot(s)ides	Thymine	10,05	255
Amino acids	Tryptophan	18,88	273
Amino acids	Tyrosine	16,26	218
Nucleot(s)ides	Uracil	9,23	241
Amino acids	Valine	7,59	144
Nucleot(s)ides	Xanthine	21,80	353
